# Transient lensing from a photoemitted electron gas imaged by ultrafast electron microscopy

**DOI:** 10.1038/s41467-020-16746-z

**Published:** 2020-06-12

**Authors:** Omid Zandi, Allan E. Sykes, Ryan D. Cornelius, Francis M. Alcorn, Brandon S. Zerbe, Phillip M. Duxbury, Bryan W. Reed, Renske M. van der Veen

**Affiliations:** 1 Department of Chemistry, University of Illinois at Urbana-Champaign, Urbana, IL 61801 USA; 2 Materials Research Laboratory, University of Illinois at Urbana-Champaign, Urbana, IL 61801 USA; 3 Department of Physics and Astronomy, Michigan State University, East Lansing, MI 48824 USA; 4 Integrated Dynamic Electron Solutions, Inc. (IDES), Pleasanton, CA 94588 USA; 5 Department of Materials Science and Engineering, University of Illinois at Urbana-Champaign, Urbana, IL 61801 USA

**Keywords:** Imaging techniques, Laser-produced plasmas

## Abstract

Understanding and controlling ultrafast charge carrier dynamics is of fundamental importance in diverse fields of (quantum) science and technology. Here, we create a three-dimensional hot electron gas through two-photon photoemission from a copper surface in vacuum. We employ an ultrafast electron microscope to record movies of the subsequent electron dynamics on the picosecond-nanosecond time scale. After a prompt Coulomb explosion, the subsequent dynamics is characterized by a rapid oblate-to-prolate shape transformation of the electron gas, and periodic and long-lived electron cyclotron oscillations inside the magnetic field of the objective lens. In this regime, the collective behavior of the oscillating electrons causes a transient, mean-field lensing effect and pronounced distortions in the images. We derive an analytical expression for the time-dependent focal length of the electron-gas lens, and perform numerical electron dynamics and probe image simulations to determine the role of Coulomb self-fields and image charges. This work inspires the visualization of cyclotron dynamics inside two-dimensional electron-gas materials and enables the elucidation of electron/plasma dynamics and properties that could benefit the development of high-brightness electron and X-ray sources.

## Abstract (web summary)

Excited charge carriers, such as photoelectrons, play an important role in fundamental and technological fields. Here the authors employ an ultrafast electron microscope to directly visualize the cyclotron oscillations and oblate-to-prolate shape change of a photoemitted electron gas from a laser-excited copper surface.

## Introduction

The non-equilibrium dynamics of charge carriers (electrons/ions/holes) plays a crucial role in a vast range of fundamental and technological fields, including chemistry, solid-state physics, plasma physics, and high-brightness electron sources. Carrier motion often unfolds on ultrafast time scales and requires tools that can directly visualize the dynamics with appropriate spatial and temporal resolutions, i.e. Ångstroms–micrometers (Å–μm) and femtoseconds–nanoseconds (fs–ns), respectively. In this regard, ultrafast electron microscopy (UEM) has recently emerged as a powerful technique for the study of ultrafast photoinduced processes in nanoscale systems^1–15^. The material is excited by a short fs–ns laser pulse, which is followed by a similarly short electron pulse that probes the ensuing dynamics by means of imaging, diffraction, or spectroscopy inside a transmission electron microscope (TEM).

Here, we use UEM to visualize the ultrafast evolution of a hot three-dimensional (3D) photoemitted electron gas under a static magnetic field in real time and real space. Confined electron gases^16^ can exhibit intriguing properties such exceptionally high electron mobilities^17^, quantum Hall effects^18,19^, Shubnikov–de Haas oscillations^20^, anomalous de Haas–van Alphen effects^21^, and superradiant damping^22^. Understanding and controlling these phenomena are of fundamental importance in diverse fields of quantum science and technology^23,24^. For example, two-dimensional (2D) electron gases at semiconducting heterointerfaces or in 2D materials, which are subject to an external magnetic field, have been studied by frequency- and time-domain THz spectroscopies^18,22,25–27^. An electron gas in a uniform magnetic field executes circular Larmor orbits in a plane perpendicular to the magnetic field. Transitions between the eigenstates (Landau levels) of electron gases confined by a magnetic field are called cyclotron resonances, whose frequencies, line widths, and decays have been used to determine band structures, effective masses, carrier densities, mobilities and scattering times in semiconducting materials^22,26,28–31^. Quantum effects arising from Landau levels are dominant when the mean thermal energy of the gas is smaller than the energy level separation, which means experiments are often performed at low temperatures and under strong magnetic fields.

The proof-of-principle UEM experiments on 3D electron gases in uniform magnetic fields presented in this work represent the first step towards the visualization of cyclotron dynamics inside materials, in particular 2D electron-gas systems such as GaAs/AlGaAs^19,26,32^ or graphene^27,33,34^. In contrast to frequency- or time-domain THz/microwave spectroscopic investigations, performing such experiments inside an ultrafast electron microscope would enable spatially resolving photoexcited carrier density variations, similar to previous scanning probe microscopy experiments^32,35–37^ but with fs–picosecond (ps) temporal resolution. Furthermore, the capability to image and temporally resolve photoemitted carriers is highly relevant in the plasma physics community^38–41^, and for the development and characterization of high-brightness electron sources for fourth-generation X-ray facilities or ultrafast electron diffraction and microscopy setups^42–50^. The analytic model we develop here allows rough approximation of the number of electrons in the photoemitted gas, which is directly correlated with the electron lens magnification, as well as their velocity spread. Systematic variations of the laser fluence and wavelength, and adding a bias to the sample, will enable obtaining valuable insight into the electron emission process and the subsequent processes that affect electron beam properties such as emittance.

## Results

### Direct imaging of electron cyclotron oscillations on the picosecond time scale

**Fig. 1:**
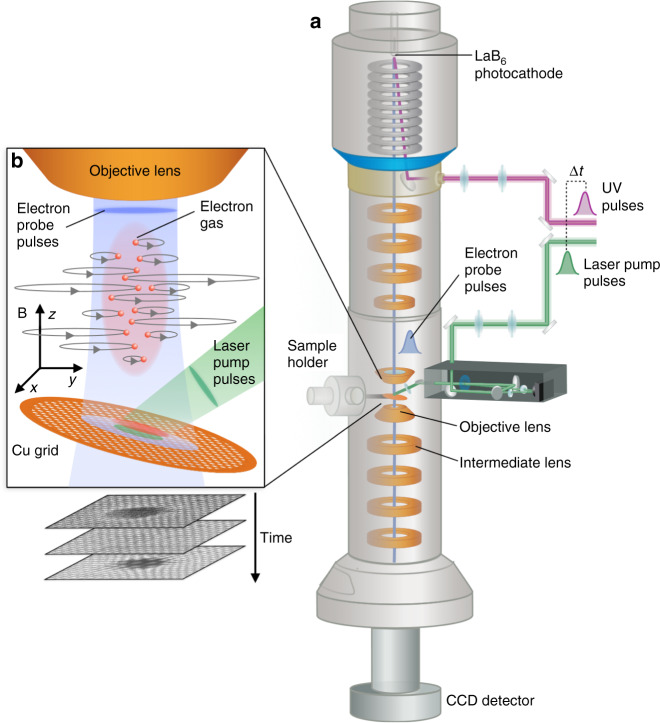
Schematic of the ultrafast electron microscope used for imaging photoemitted electron gas dynamics. **a** Short probe electron pulses (~500 fs, 490 kHz, 300 keV) are generated by impinging a UV (256 nm) laser beam onto a LaB_6_ photocathode. Pump laser pulses (~200 fs, 528 nm, 490 kHz, ~30 mJ/cm^2^) are focused onto the Cu grid sample inside a modified TEM. b A hot electron gas (red), created by means of two-photon emission, acts as a diverging lens to the probe electrons. After initial Coulomb explosion, the electron gas executes cyclotron oscillations (gray orbits) inside the magnetic field (**b**) of the objective lens, which are resolved by changing the relative timing of the pump and probe pulses (Δ*t*). The strength of the intermediate lens in the TEM can be tuned to obtain different imaging conditions. The sample is tilted by 15^o^ towards the pump laser in order to minimize the laser footprint on the sample.

We performed our experiments using a modified environmental TEM operating at 300 keV (Fig. 1a), which is interfaced with a high repetition rate, fs laser system (see Methods section for more details). Laser pump pulses (~200 fs, 528 nm, ~30 mJ/cm^2^) are guided onto the sample using a mirror/lens system that inserts into the energy-dispersive spectroscopy (EDS) port of the TEM. Short probe electron pulses (<1 ps) are generated by impinging a UV laser beam onto a LaB_6_ photocathode. The laser pump and electron probe pulses are precisely synchronized in time at a repetition rate of 490 kHz and their relative delay is adjusted by means of an optical delay stage. In this way, we record real-space movies of the charge density dynamics after laser excitation with an integration time of 1 s (5  × 10^5^ shots) per frame. The sample consists of a tilted 3000 mesh copper grid with ~4.5 μm hexagonal holes, which are significantly smaller than the laser footprint of 22 × 36 μm (full width at half maximum, FWHM) on the sample. Since the photon energy (2.35 eV) is half the work function of copper (4.5–4.7 eV^51^), photoelectrons are emitted in the two-photon absorption regime^45,52^. The laser fluence is low enough that ablation and (neutral) plasma formation can be discarded^53^.

**Fig. 2:**
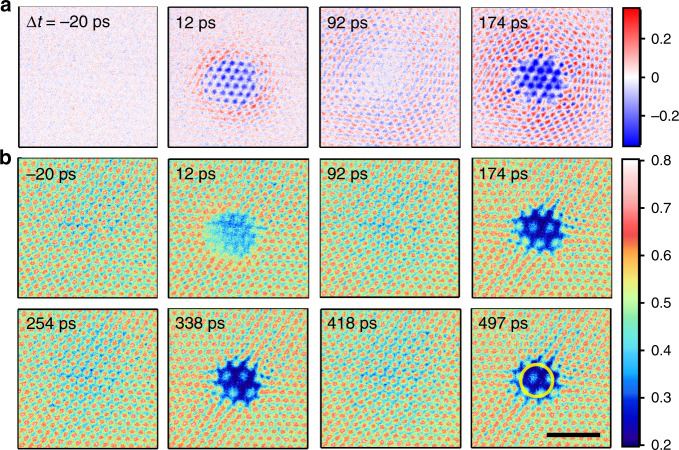
Transient electron-gas lensing images. Series of 3000 mesh copper grid images (**b**) and difference images (**a**) extracted from a ps-resolved UEM movie (528 nm, 200 fs, 30 mJ/cm^2^ laser excitation) with an objective lens current of 0.7 A. The time delays correspond to the first few local maxima and minima in the ROI difference intensity trace in Fig. 3. The difference images were generated by subtracting an averaged image before time zero (Δt = 0). A typical region-of-interest (ROI) circle that is used to make plots of the intensity changes due to lensing is indicated in the last frame. The scale bar at the bottom right is 50 μm and applies to all images. The intermediate lens current was set to 0.65 A.

**Fig. 3:**
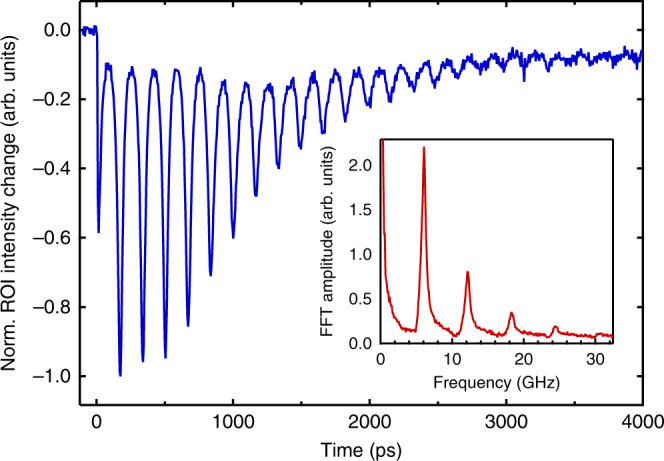
Region-of-interest (ROI) intensity analysis of transient lensing dynamics. ROI difference intensity (relative to before time zero) as a function of time delay. A typical ROI is denoted in Fig. 2b (different data set). The inset shows the FFT of the trace, with a fundamental frequency of 6.05 GHz (i.e. *T* = 165 ps). The objective lens current was set to 0.7 A and the intermediate lens current was set to 0.65 A.

Figure 2a, b shows a set of copper grid (difference) images that is extracted from a ps-resolved UEM movie (see Supplementary Information, SI, movies S1 and S2). Upon laser excitation, a prompt depletion of intensity in the probe image occurs (12 ps frame), which lasts for approximately 50 ps. The subsequent dynamics is characterized by localized, periodic barrel distortions of the grid images at intervals of ~165 ps, with almost no changes in between resonance peaks. A region-of-interest (ROI) analysis for a similar data set (Fig. 3) shows that these recurring image distortions continue for more than twenty cycles over a time span of as much as ~4 ns, and with a fundamental frequency of 6.05 GHz as shown by the fast Fourier transformation (FFT) of the ROI trace. Except for higher harmonics resulting from the pointed shape of the resonance peaks, no other frequencies are contained in the data. The barrel distortions are due to a transient lensing effect of the photoemitted electron gas, whose transverse electric field causes a deflection of the probe electron pulses leading to a magnification in the projected image on the detector. The photoemitted electron cloud therefore acts as a 3D diverging lens to the probe electron pulses.

**Fig. 4:**
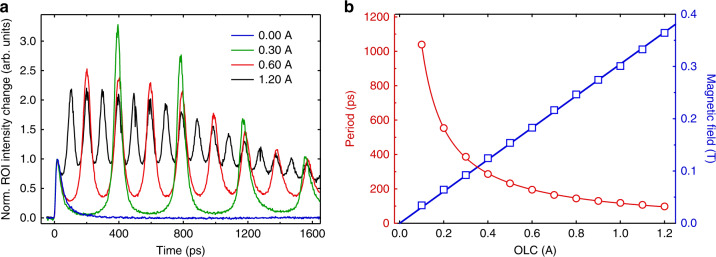
Dependence on the objective lens current. **a** ROI difference intensity traces as a function of objective lens current (OLC). The traces have been normalized to the height of the first peak ~12 ps after photoexcitation. The intermediate lens current was set to 1.1 A. Note that there are no oscillations for an OLC = 0 A. **b** Cyclotron oscillation period (red, left axis) as a function of the OLC. Using the cyclotron period formula *T* = *2πm*_e_*/eB*, the corresponding magnetic field at the sample position is calculated (blue, right axis). The latter corresponds well with data from the manufacturer (see SI S1). The solid blue line is a linear fit to the data.

The electron gas is subject to a static magnetic field (**B**) along the electron-optical axis which is imposed by the objective lens (upper and lower pole piece) of the TEM. This field is relatively weak due to the fact that we operate the TEM in low-magnification mode. Under the influence of the Lorentz force **F** = *q***v** × **B**, the electrons gyrate around the magnetic field axis, with an electron cyclotron period of *T* = 2*πm*_e_/*eB*, where **v** is the velocity vector of the electrons, *m*_e_ is their mass, and *e* is the elementary charge (Fig. 1b). The (non-relativistic) radius of gyration for each electron is given by *r* = *v*_T_*m*_e_/*eB*, with *v*_T_ the transverse (*x*, *y*) velocity component in the direction perpendicular to the magnetic field (*z*). Each electron therefore circulates with a different radius, depending on its initial velocity, but all electrons that are not absorbed by the grid reconvene to their initial positions in the *x*, *y*-plane after a full cyclotron period *T*. Slightly before this point in time, the collective width of the electron gas reaches a minimum and the transverse electric mean-field maximizes resulting in a pronounced transient lensing effect that is observed in the probe image. While the magnetic field confines the electron gas in the transverse direction, the longitudinal dynamics is affected by the *z*-velocity profile and the boundary conditions at the surface of the copper grid. We show later that this anisotropic confinement causes a pronounced oblate-to-prolate shape transformation of the 3D electron gas and a large concurrent increase of the lensing strength on the time scale of ~100 ps. Figure 4a shows ROI difference intensity traces recorded at various objective lens currents (OLC). Using the cyclotron period formula above and a FFT analysis of the ROI traces, we constructed a plot of the OLC versus cyclotron period and magnetic field (Fig. 4b). A linear relationship between OLC and magnetic field is obtained, which matches data from the TEM manufacturer reasonably well (see SI1). The ROI intensity trace for OLC = 0 A (no magnetic field) merely shows the first peak, as expected.

We note that deflection effects due to transient electric fields from photocreated electron plumes have been observed previously in ultrafast electron diffraction and microscopy setups^38,39,54–60^. However, we report for the first time a detailed study of the space-charge dynamics in the presence of a magnetic field and the consequent changes in the image under various experimental conditions.

### Dependence on the imaging conditions and electron-gas astigmatism

**Fig. 5:**
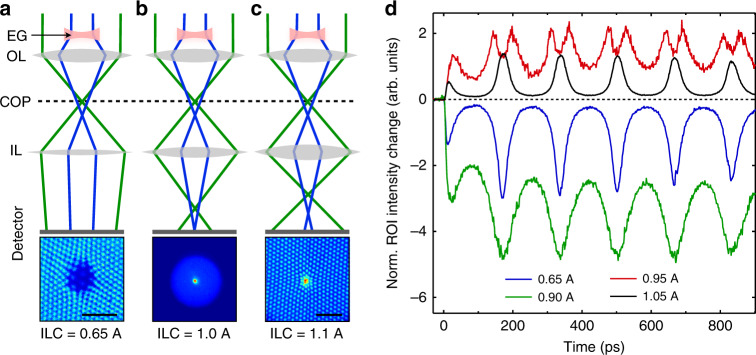
Dependence on the intermediate lens strength. **a**–**c** Image conditions with various intermediate lens currents (ILC): **a** 0.65 A, underfocus regime, barrel distortion, **b** 0.95 A, in-focus regime, **c** 1.1 A, overfocus regime, pincushion distortion. The corresponding images are shown at the bottom (Δ*t* = 173 ps, OL current 0.7 A). Since the electron gas (EG, red) acts like a diverging lens, the probe electrons that pass through the gas (blue) are focused by the objective lens (OL) after/below the probe electrons that are not affected by the electron lens (green). COP = crossover plane. The scale bars are 50 μm (no scale bar for image in **b**, since the grid is not visible). In this figure, all the projection lenses after the sample are replaced by one equivalent lens (labeled IL). Only the IL strength was changed. The copper grid is not shown for simplicity. d ROI difference intensity traces for various ILC values (OL current 0.7 A).

The transient lensing effects are only visible in the images if the projection lens system of the TEM is set to out-of-focus image conditions. This is a consequence of the geometry. Because the laser impinges on the grid from above, and because the fill fraction of the grid is quite high (66%), nearly all the photoemitted electrons will be above the grid. The grid itself will act as an electrostatic boundary condition preventing the space-charge electric fields from penetrating significantly below. Thus, the lensing effect of the electron cloud will deflect electrons radially before they strike the grid but will have essentially no effect on the grid pattern itself or on the focusing action produced by the TEM lenses below the grid. In a focused real-space image, the post-sample lenses map the (*x*, *y*) spatial positions of electrons as they emerge from the back of the sample linearly onto the camera, suppressing information about the angles of the electron trajectories. Thus, an in-focus image should just produce an image of the grid, as we observe. To detect the space-charge lensing effect, we defocus the imaging system so that the resulting image is a linear combination of the spatial and angular coordinates of the electrons emerging from the back of the sample. Because the OLC is an important parameter for the space-charge dynamics, we instead adjust the current of the first intermediate lens (IL), i.e. the first lens after the objective lens (see Fig. 1a). We recorded lensing movies (see movies S2–S4) for a range of IL excitation strengths. In this way, we are able to tune the electron-gas lensing effect from a magnifying, barrel image distortion for low IL excitations (Fig. 5a), to a demagnifying, pincushion image distortion for high IL excitations (Fig. 5c). For intermediate IL strength (Fig. 5b) we can image the post-sample crossover of the objective lens onto the detector. Thus, the defocused images reveal the shift in the post-sample crossover caused by the lensing effect of the electron gas, manifesting as a magnification or demagnification of the affected region relative to the rest of the grid.

**Fig. 6:**
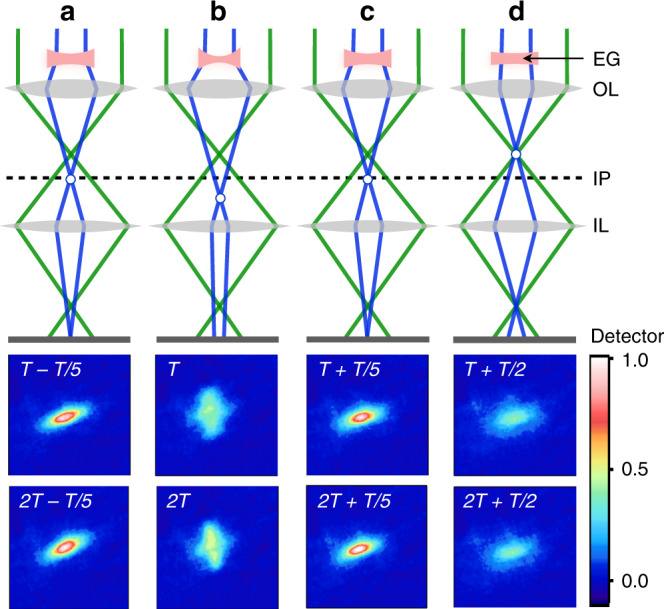
Double-peak structure in ROI difference intensity traces and astigmatism of the electron-gas lens. Schematics of the electron gas (EG, red) acting as a biconcave lens while it reaches its largest lensing strength (**b**). The intermediate lens (IL) current (0.95 A) is set such that the image plane (IP) denoted by the dashed line is projected onto the detector. Depending on the strength of the EG lens, the crossover point (open circle) lies either on (**a**, **c**), below (**b**), or above (**d**) the IP. Under these conditions the ROI difference intensity shows a double-peak structure (see e.g. Fig. 5d, different data set), with local maxima appearing at approximately *nT* ± *T*/5(*n* = 0, 1, 2,…), *T* being the cyclotron period as defined in the text. The focal point is astigmatic due to the elliptical shape of the EG lens, as is seen by the rotation of the elongated focal point as it moves through the IP. The objective lens (OL) current was set to 0.7 A, with *T* = 165 ps.

Since the electron cloud acts like a diverging lens, the probe electrons that pass through the cloud have a longer effective focal length after the objective lens than the probe electrons that pass further away and are not affected by the electron gas. If the IL current (ILC) is set to a value such that a plane between the post-sample crossover of the unaffected probe electrons and the post-sample crossover of the lens-affected probe electrons is imaged onto the detector (Fig. 6), the ROI difference intensity trace shows a double-peak structure as shown in Fig. 5d for ILC = 0.95 A. A representative set of difference images recorded under such conditions is shown at the bottom of Fig. 6 (ILC = 0.95 A, OLC = 0.7 A), from which we see that the focal point is significantly astigmatic, i.e. the elongated shape of the spot rotates by approximately 90° as it moves through focus (see movie S5). We ascribe this astigmatism to the fact that the electrons are emitted from a slightly (15°) tilted grid surface. Their initial velocity distribution is aligned perpendicular to the sample surface, which translates into an increased transverse velocity component in the tilt direction relative to the electron-optical axis (*z*). In addition, the shape of the photoemitted electron gas is not perfectly circular in the *x,y*-plane due to shallow angle (37°) between the excitation laser and the sample surface, and the ensuing elliptical footprint of the laser on the sample. The astigmatism is not visible in all data sets, as it can be compensated by the condenser stigmator correction lenses inside the TEM. The tilt of the sample does not affect the ROI intensity dynamics at low or high IL currents (see SI2).

### Analytical model for electron density evolution and determination of the transient focal length

In order to quantitatively describe the evolution of the 3D electron gas, we developed an analytical model that allows us to estimate the velocity spread of the photoemitted electrons, the number of electrons in the gas, as well as the time-dependent focal length of the electron lens. It is known that the velocity profile of one-component plasma systems expanding under their mutual repulsion, known in the literature as Coulomb explosion, is largely determined by the early dynamics of the expanding bunch where the density, and hence the force between particles, is largest^43,61,62^. Therefore, we focus our analysis on the time range >50 ps once the velocity profile has largely been established (see below for numerical simulations that confirm this). In this regime we describe the electron gas as a cylindrically symmetric Gaussian charge distribution with a time-dependent transverse radius *σ*_T_(*t*), and an axial radius *σ*_*z*_(*t*) (*σ* denotes standard deviation). While the evolution of *σ*_*z*_ is essentially linear in time due to the almost free (linear) expansion of the gas in this direction, the transverse radius is affected by the Lorentz force **F** = *q***v** × **B**. Solving the equations of motion (see SI3.1 for details), we obtain the periodic dependence for the transverse radius of the electron gas

1 $$\sigma _{\mathrm{T}}\left( t \right) = \sqrt {2\left( {\frac{{\sigma _v}}{\omega }} \right)^2\left( {1 - \cos \left( {\omega t} \right)} \right) + \sigma _r^2} ,$$

where *ω* = *eB*/2*m*_*e*_ is the cyclotron angular frequency, *σ*_*v*_ is the velocity spread in the transverse direction, and *σ*_*r*_ is the minimum transverse radius of the electron gas. Equation (1) shows that at times *t* = 2*nπ*/*ω* (*n* = 0, 1, 2,…) the transverse radius reaches its smallest value *σ*_*r*_, and the electron number density concurrently maximizes. These periodic electron density peaks are responsible for the transient lensing effects in the probe images (e.g. Fig. 2).

Using Maxwell–Gauss law, we derive an expression for the radial electric field Ɛ_*r*_(*t*) associated with the cylindrical Gaussian charge density (see SI3.2) and find $\varepsilon _r \propto r/\sigma _{\mathrm{T}}^2$ in the limit *r* < *σ*_T_, i.e. close to the center of the electron cloud the field is linear with radius *r* which imparts the lensing effect on the probe electrons. Assuming that the duration of the interaction between the relativistic probe electrons and the electron gas is short compared to the evolution time scale of the gas, and also using the thin-lens approximation, we derive the focal length of the electron gas as (see SI3.3)

2 $$f_{{\mathrm{EG}}}\left( t \right) = - \frac{{2\left( {2\pi } \right)^{\frac{3}{2}}{\it{\epsilon }}_0\gamma v_z^2m_{\mathrm{e}}}}{{Ne^2}}\left[ {2\left( {\frac{{\sigma _v}}{\omega }} \right)^2\left( {1 - \cos \left( {\omega t} \right)} \right) + \sigma _r^2} \right],$$

where ${\it{\epsilon }}_0$ is the vacuum permittivity, γ = 1.6 is the relativistic Lorentz factor, *v*_*z*_ = 2.3·10^8^ m/s is the velocity of the 300 keV probing electrons, and *N* is the number of electrons in the cloud. The focal length is inversely proportional to the number of electrons in the cloud, and it is negative as is expected for a diverging lens. Furthermore, it follows a similar periodic dependence as the transverse radius *σ*_T_(*t*), with minima in absolute focal length $\left| {f_{{\mathrm{EG}}}\left( t \right)} \right|$ (maxima in lensing strength) occurring at times *t* = 2*nπ*/*ω* (*n* = 0, 1, 2,…). The electron cloud always deflects the probe electrons, but the deflection strength varies over time. Between the cyclotron peak maxima electrons are well spread in the transverse plane. The maximum width of the cloud depends on the electron’s velocity spread and the magnetic field strength. The larger the velocity spread, the larger the maximum radius of the cloud, and hence the lower the electric field and the smaller the ROI signal between the peaks. For this reason, the ROI signal does not go to zero between the cyclotron peaks, as seen in Fig. 3.

Equation (2) shows that the number of electrons, the velocity spread, and the minimum transverse radius are needed to obtain values for the focal length of the electron gas. In the absence of any extracting field, the velocity spread of the electron cloud primarily comes from the photoemission process, Coulomb interactions at early times, and the dipole field between the electrons and their positive image charge^43,49,61–65^. The number of electrons in the electron gas is determined by the laser fluence, wavelength, and photoemission quantum yield and by the absorption of electrons by the copper grid. Since these quantities are not known a priori, we estimate them from our data. In SI3.4 we demonstrate that the change in ROI intensity is inversely proportional to the electron-gas focal length when the intermediate lens is highly excited, i.e. for ILC > 1 A. This allows us to fit the ROI difference intensity trace for an ILC of 1.1 A to the function $1/f_{{\mathrm{EG}}}\left( t \right) = Ae^{ - t/\tau }/\sigma _{\mathrm{T}}^2\left( t \right)$, where fitting parameter *A* encompasses the number of cloud electrons and other unknown parameters that describe the strength of the lenses in the projection system of the TEM (see SI4 for fitting details). The exponential factor *e*^−*t*/*τ*^, with fitting parameter τ, phenomenologically describes the slow decay of the electron lens strength due to the loss of electrons over time and dephasing. The latter occurs due to the non-uniformity of the magnetic field for distances >1 mm away from the sample surface. The magnetic field strength drops by 20% at ~1.5 mm above and below the eucentric sample plane (see SI1). As the electron cloud expands along the magnetic field direction, the electrons on the top part of the cloud will therefore oscillate with a lower frequency than electrons that stay relatively close to the grid. The overall effect is a reduction of the oscillation contrast, i.e. the ratio of absolute ROI peak maximum to minimum (between peaks), which is roughly modeled by the exponential decay factor. We set the minimum transverse radius to $\sigma _r = 12/\sqrt 2$ μm, which is obtained from the experimental laser spot size of ~28 μm FWHM or *σ* = 12 μm (geometric average of major and minor axes of elliptical footprint), and considering that the electrons are emitted through a two-photon process that scales quadratically with photon intensity. For concreteness, here we use a simplified model of the TEM post-sample lensing system allowing us to produce order-of-magnitude estimates of both *f*_EG_ and the number of electrons *N*~10^5^ in the cloud (see SI3.4–5).

**Fig. 7:**
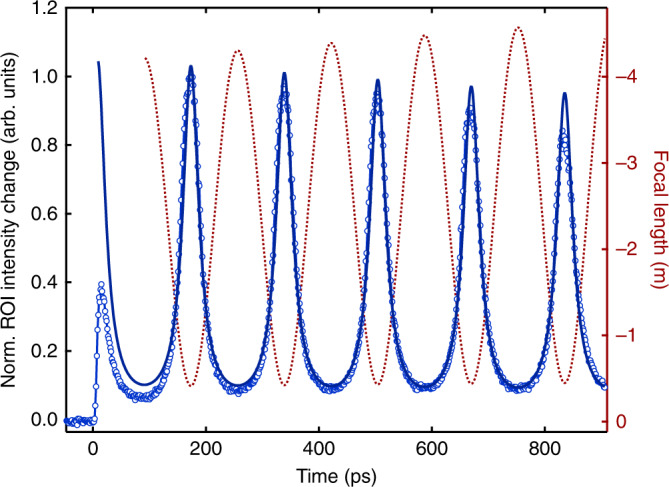
Comparison between experimental and theoretical electron cyclotron dynamics. The ROI difference intensity trace for ILC = 1.1 A (blue circles, left axis) is fitted to the function $1/{\it{f}}_{{\mathrm{EG}}}(t) = {\it{Ae}}^{ - {\it{t}}/{\it{\tau }}}/{\it{\sigma }}_{\mathrm{T}}^2\left( {\it{t}} \right)$ (dark blue line), where *A* and τ are phenomenological fitting parameters encompassing the time-dependent number of electrons in the cloud and TEM-specific lens settings. *σ*_T_ is the time-dependent transverse radius of the electron gas, which depends on the velocity spread *σ*_*v*_, the minimum radius *σ*_*r*_, and the cyclotron angular frequency ω (all fitted to the data). The fitting range is 100–900 ps, i.e. beyond the regime where Coulomb interactions are significant. The corresponding focal length of the electron gas, *f*_EG_, is plotted in red (right axis), which is only an order-of-magnitude estimate due to the approximations in the model (see text).

The resulting fit is shown in Fig. 7, together with the corresponding electron-gas focal length. Interestingly, the focal length magnitude varies by about a factor of ten, ranging from ~0.5 m at the lensing maxima to ~4–5 m in between cyclotron resonances. From the fit we are able to determine the angular frequency *ω* = 37.97 ± 0.01 GHz, and the transverse velocity spread *σ*_*v*_ = 4.91±0.01·10^5^ m/s (the standard deviations on the fit parameters do not reflect the inaccuracies of the model itself), the latter of which largely determines the width of the resonance peaks (see SI5). The decay constant τ is on the order of several ns, but could not be determined with high accuracy due to the limited fitting window. We note that the quantities *σ*_*v*_ and *σ*_*r*_ are not expected to vary with time because the magnetic field does no work and we are treating the self-interaction as negligible resulting in no appreciable electric field. Furthermore, the good agreement between the model and the data indicates that space-charge effects play a negligible role in this time regime (>100 ps). On the other hand, the amplitude of the ROI intensity change at the first peak predicted by theory is much larger than the experimentally measured amplitude. In fact, the ratio of the first-to-second peak amplitude is consistently ~0.3–0.5, regardless of the objective or intermediate lens strength or slight variations in the ROI radius (except for OLC = 0 A). Clearly, we need to include other elements such as Coulomb self-fields and the copper grid in order to quantitatively describe the early dynamics <100 ps.

### Numerical *N*-body simulations of the photoemitted electron cloud dynamics in the presence of Coulomb interactions and a copper grid

**Fig. 8:**
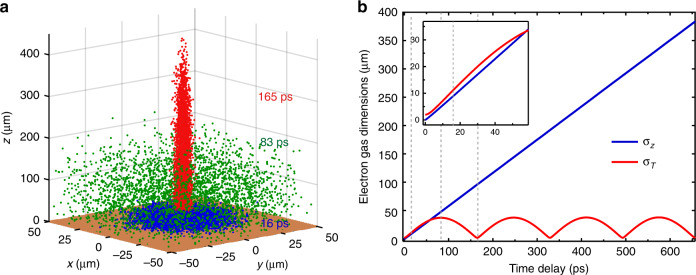
Results from numerical N-body simulations. **a** Snapshots of the electron distribution (10,000 electrons) taken at three different time delays. The copper grid is schematically shown in brown. **b** Electron gas dimensions (standard deviations) in the transverse (*x*, *y*) and axial (*z*) directions as a function of time, extracted from the N-body simulation with *B* = 0.22 T. The inset shows a zoom into the dynamics during the first 18 ps. The vertical lines indicate the time delays at which the snapshots in a were taken.

In order to get a holistic picture of the electron dynamics, including Coulomb interactions within the electron cloud, we performed numerical *N*-body simulations for a realistic charge density that is subjected to a uniform, static magnetic field. Details of the numerical simulations are given in the Methods section. Briefly, with small time increments, we calculate the Lorentz force, **F** = *q*(**E** + **v** × **B**), and resulting displacement of each electron inside an initially 3D Gaussian oblate (2 × 2 × 0.01 μm^3^) electron cloud with 10^4^ electrons. These parameters were chosen in order to approximately match the initial charge density in the experiment, obtain the same order of magnitude velocity spread, and reproduce the ratio of the first-to-second peak intensity without absorption of the majority of electrons due to the strong electric field between the cloud and the grid. The electromagnetic forces arise from self-Coulomb fields, the external magnetic field, as well as positive image charges due to the existence of the copper grid that acts as a planar conductor held at zero potential (connected to the body of the TEM). Any positive residual bulk charges will be screened on the time scale of the metal plasma oscillation (sub-fs) and move to the surface to form a nm-thin charge layer that is negligible on the scale of the simulations (see SI8). There is therefore no electric field below the grid. We assume that electrons that hit the grid will be absorbed and hence omitted from the rest of the calculation. Snapshots taken from a simulation with *B* = 0.22 T at three time delays are superimposed in Fig. 8a (full movie S6). The frame at 16 ps after photoexcitation shows a flat electron distribution close to the copper grid, that has already significantly expanded due to Coulomb explosion of the gas during the first few ps. The distribution in the intermediate 83 ps frame, which corresponds to the first minimum in the electron cloud density, is homogeneously spread out over tens of μm in all (*x, y, z*) directions. Finally, for the frame at 165 ps, which corresponds to the first cyclotron resonance peak, the electron gas regains its narrow transverse size, but it is severely elongated along the *z*-axis.

The corresponding radial and axial standard deviations of the electron cloud as a function of time are plotted in Fig. 8b. Within 165 ps, the electron gas morphs from an oblate (pancake-like) distribution, with an aspect ratio of $\sigma _z/\sigma _{\mathrm{T}} \simeq 0.005$ and charge density of 40 mC/cm^3^ (per $\sigma _{\mathrm{T}}^2\sigma _z$), into a prolate (cigar-like) shape with an aspect ratio of $\sigma _z/\sigma _{\mathrm{T}} \simeq 50$ and density of 2 μC/cm^3^. This corresponds to a factor of 20 × 10^3^ decrease in charge density, which is due to both the shape transformation as well as absorption of electrons by the grid (see SI6). For time delays >4 ps, the gas linearly expands along the *z*-direction with a velocity of ~6 × 10^5^ m/s, while it is refocused to a little past its initial radial size in the *x,y*-plane at intervals of *T* = 165 ps. At a time delay of 3 ns, the axial size of the electron gas reaches ~1.5 mm. The decay of the lensing signal and damping of the oscillations is therefore ascribed to a combination of continuing absorption of electrons by the copper grid (which is only ~10% over ~6 ns, see SI6), as well as a change of the cyclotron frequency due to the decrease of the axial magnetic field for distances >1 mm from the sample (see SI1). Absorption of electrons by the upper pole piece of the objective lens can also not be neglected on these time scales. Both latter effects are not considered in the simulation. The slow time scale of these loss/damping processes explains the exceptionally long lifetime of the cyclotron oscillations. Due to Coulomb explosion, the electron velocity distribution (mean, spread) changes abruptly during the first few ps after photoexcitation, but it reaches a plateau for time delays >50 ps (see SI7). The time-averaged transverse velocity spread from the simulation for times >50 ps, ~7 × 10^5^ m/s, is in reasonable agreement with the velocity spread obtained from fitting the analytical model to the ROI intensity data (*σ*_*v*_ ~ 5 × 10^5^ m/s, Fig. 7). This agreement confirms that the initial conditions of the simulations (electron density, velocity, and velocity spread) were chosen appropriately. We refer to the 3D electron gas to be “hot”, since the electrons have a random kinetic energy distribution with typical values much larger than *k*_B_*T*, where *T* is the temperature of the source material.

The rapid oblate-to-prolate shape transformation of the electron gas, evidenced by the numerical simulations, has profound influence on its transient lensing strength. Indeed, the first peak in the ROI difference intensity traces is observed ~12 ps after photoexcitation (as opposed to just after time zero) and it is consistently lower in amplitude than the second peak. Qualitatively, we can attribute this to two things: first, the oblate shape of the electron gas at early times leads to a small transverse electric field component, and therefore a reduced impulse on the probing electrons. Second, the transverse electric field component is further reduced by the positive image charges, effectively creating a parallel-plate capacitor at early times. As the electron bunch expands during the first few ps, the electron gas adopts a prolate shape elongated along the *z*-axis, and the effect of the image charges is reduced since the electrostatic dipole force along *z* scales with ~1/*d*^2^ where *d* is the distance between the two charges (see SI8). The transverse electric field component, and therefore lensing strength, increases concurrently, despite the absorption of electrons by the grid.

We would like to stress that even in the absence of a magnetic field, an oblate-to-prolate shape transformation is expected for the electron cloud, although it will not be as pronounced. This relates to the photoemission process itself: electrons are impulsively (within ~150 fs) expelled from the copper surface with a lateral dimension determined by the laser spot size on the sample; the initial longitudinal size of the bunch is very small. The photocreation process on ultrafast time scales therefore always leads to a pancake-like (oblate) electron bunch. Under the influence of self-Coulomb fields, the bunch rapidly (in a few ps) expands, which affects the longitudinal dimension more than the lateral dimension^43,65,66^. Even in the absence of an extraction field, this leads to a change in shape of the bunch from oblate to prolate. The magnetic field, however, confines the electrons only in the lateral dimension, while the electrons continue to expand along the longitudinal dimension. This enhances the shape transformation significantly, resulting in the very large change of aspect ratio of a factor of ~10^4^. The shape change is not due to Coulomb interactions when electrons reconvene along the *z*-axis during the cyclotron oscillations; such interactions are negligible on time scales beyond ~50 ps. The shape transformation is thus a general feature for electron bunches that are created by ultrafast laser pulses (pulse duration ≪ cyclotron period) and that are subject to a magnetic field (nearly) perpendicular to the surface. However, for other parameters, such as longer laser pulse duration^65^ (tens of ps) or higher magnetic field strengths, one could achieve other shape and lensing dynamics.

**Fig. 9:**
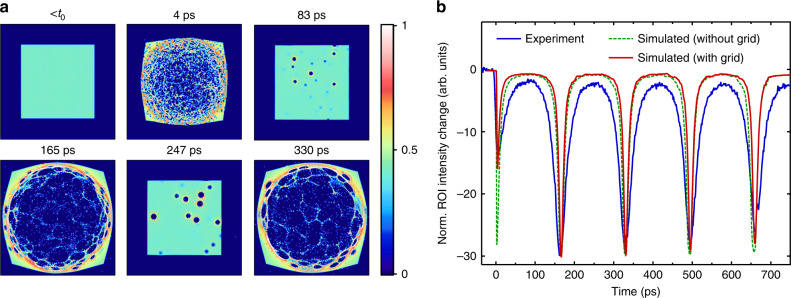
Probe image simulations. **a** Simulated grid images based on snapshots from the numerical *N*-body simulation. The sides of the images are 24 μm. Each electron on the detector is represented by a Gaussian kernel with a width of three pixels. **b** Simulated and experimental ROI difference intensity traces. The traces have been normalized to the second peak at 165 ps. The dashed green curve represents a calculation without a copper grid (no absorption of electrons, no image charges). The red curve includes the grid.

In order to confirm this interpretation on a more quantitative basis, we simulated the UEM lensing movies by sending a regular grid of relativistic probe electrons through each frame of the *N*-body simulation (see Methods section for details). Here, we neglect Coulomb interactions between probe electrons, as well as any perturbations of the electron gas by the probe electrons. Representative snapshots of these probe simulations are shown in Fig. 9a (full movie S7), which can be compared to the experimental movie frames in Fig. 2b. All features are reproduced well, including the depletion of the probe intensity in the center, a bright ring around the depletion area in the first peak, a delay in reaching the first maximum lensing strength at Δ*t* = 4 ps after photoexcitation (see SI9, ~12 ps in the experiment), as well as a profound magnification of the grid images at the cyclotron resonance peaks (Δ*t* = 165 ps, 330 ps). The lensing is much stronger at the cyclotron resonance peaks, when the electron gas adopts a prolate shape, than at the first peak, when it has a more oblate shape. Corresponding ROI difference intensity traces, with and without the copper grid included, are shown in Fig. 9b, together with an experimental ROI trace taken at low IL currents. The agreement is satisfactory, in particular the ratio of the first and second peak amplitudes is reproduced very well, as well as the shape of the resonance peaks. The discrepancy in width of the peaks is assigned to differences in the electron velocity spread and the number of electrons, which are difficult to get right without explicitly including the photoemission process itself. We emphasize that the first-to-second peak amplitude ratio is only simulated well if the copper grid is included in the simulation. This shows that the image charges, as well as the absorption of electrons by the grid during the Coulomb explosion, play a significant role in the dynamics <50 ps. The discrete depletion areas in Fig. 9a are due to the granular nature of the electron cloud in the *N*-body simulations. The simulation is run only once for a certain set of initial conditions, from which we derive our probe images. Small density variations in the electron cloud can lead to local lensing effects in the probe image. The resulting small depletion areas on the detector would be merged together if one would average the results of many *N*-body simulations, each with the same initial conditions (which is representative of our pump-probe experiment at 490 kHz). For this reason there is a discrepancy in experimental and simulated ROI intensity between the cyclotron peaks in Fig. 9b.

## Discussion

Using a home-built ultrafast electron microscope, we observed the ps-resolved cyclotron dynamics and lensing of a 3D hot electron gas created by photoemission from a copper target with intense fs laser pulses. Within 100–200 ps after photoexcitation, the gas undergoes an oblate-to-prolate shape transformation with a change in aspect ratio of a factor of 10^4^, and subsequent transverse expansions and contractions due to the gyration of individual electrons around the static magnetic field axis in the microscope. The cigar-shaped electron cloud acts as a diverging lens to the probe electrons, with focal lengths ranging from ~0.5–5 m during one cyclotron oscillation. We show that the observed lensing is dominated by a cooperative mean-field effect, as opposed to particle-particle scattering of individual probe and cloud electrons. Specifically, the granular nature of the electron distribution can effectively be ignored and instead can be replaced by the mean-field it creates (at least at the velocities we are considering here). Our current analytical treatment allows us to estimate the velocity spread and number of electrons in the gas, but it excludes the influence of Coulomb interactions inside the cloud, positive image charges, as well as the absorption of electrons by the grid. We performed numerical *N*-body simulations to take these effects into account, which proves to be crucial to understand and simulate the early dynamics before 50 ps. An analytical treatment including Coulomb interactions and image charges, and a more quantitative description of the TEM lensing system, will be part of future work.

These experiments inspire a plethora of future studies in at least three distinct fields. First, they present a unique way to directly visualize and characterize photoemitted charged-particle beams, which is of importance in the fields of high-brightness electrons sources for ultrafast microscopy and fourth-generation X-ray facilities, and plasma physics. Future experiments will focus on systematically investigating the dependence on laser wavelength (tuning the regime from two-photon, to one-photon and three-photon emission), and laser fluence. Furthermore, using an electrical TEM holder, one could apply a bias to the sample which enables studies below the virtual cathode limit^44,65^. Second, our work is the first step towards the investigation of charge carrier cyclotron dynamics inside photoexcited materials using UEM. Intense photoexcitation can create electron-hole plasmas, in which the electron and holes gyrate with different frequencies, direction, and spatial extent due to their distinct effective masses, charges, and diffusion lengths, respectively. This should lead to an effective (transient) spatial separation of electron and hole densities, that could be resolved by UEM. Furthermore, the implementation of quantum point contacts using a custom MEMS-based TEM holder, could enable the spatiotemporal visualization of coherent flow and magnetic focusing of charge carriers in 2D electron-gas materials^32,35–37^. Such experiments would need to be performed at low temperatures, and need materials with large carrier diffusion lengths, such as InSb, InAs, GaAs/AlGaAs, or transition metal oxides^17,26^. The electric field in solid materials is dominated by the local charge density, because the system is close to charge neutral. In contrast, in systems that are not charge neutral, such as charged-particle bunches in free space, the long-range Coulomb force leads to local electric fields that are strongly influenced by all the particles in the bunch. In this case the probe electron deflections are dominated by cooperative mean-field space-charge effects, and the scattering due to local charge inhomogeneities typical of scattering from solids is a second order effect. In considering cooperative lensing effects in electron systems confined in the solid state, such as at heterointerfaces or at surfaces, several factors arise; including scattering from the atomic structure of the hosting solid, and screening of the Coulomb interactions due to charge polarization in the solid. Though this modifies the lensing effects, especially on large length scales, qualitatively similar cooperative lensing effects, albeit much weaker, may still be expected in cases where high-density interfacial electron gases can be generated. With future developments, such as the implementation of high-frequency chopping cavities^67^ or improvements in low-temperature TEM technologies, such challenging experiments might come within reach.

## Methods

### UEM setup, experimental conditions, and data treatment

We employ a custom-modified environmental Hitachi H9500 TEM operating at 300 keV, interfaced with a high repetition rate fs laser system (Light Conversion PHAROS with ORPHEUS-F OPA) that allows excitation of the sample with wavelengths between 260 and 2600 nm and variable repetition rates up to 1 MHz. In the experiments reported here, the sample is excited using 528 nm, ~200 fs laser pulses, with fluences of ~30 mJ/cm^2^. Short probe electron pulses are generated using the photoelectric effect by impinging 256 nm, ~200 fs UV laser pulses onto a graphite guard-ring LaB_6_ photocathode with a diameter of 50 μm (Kimball Physics). Laser pump and electron probe pulses impinge the sample with a repetition rate of 490 kHz and their relative delay is controlled using an optical delay line (Aerotech). The data acquisition software is provided by IDES Inc. Typical integration times per image were 1 s, corresponding to 4.9 × 10^5^ pump/probe shots. We note that the exact temporal resolution of the setup is not known yet. However, we excite the photocathode with a low pulse energy of 16 nJ, which puts us into a regime where tens-hundreds of electrons are emitted at the photocathode, and only a few electrons will reach the sample. This so-called “single-electron” mode has previously been shown to yield instrumental response functions (IRF) that are almost entirely limited by the pump and probe laser pulse durations^68^, i.e. ~500 fs in our case. In any case, the IRF is much smaller than the cyclotron oscillation period (50–400 ps); the width of the ROI cyclotron peaks is therefore not limited by the IRF. The rise of the ROI signal intensity itself cannot be used to determine the IRF, since our simulations indicate that the rise time is prolonged in the presence of image charges (see SI9). The sample consists of a 3000 mesh copper gilder grid (SPI Supplies) with a hexagonal hole diameter of ~4.5 μm, and thickness of ~5 μm. The overall transmission is 34%. The copper grid is tilted by 15° towards the pump laser beam in order to minimize the footprint to 22 × 36 μm FWHM. The angle between the pump laser and sample plane is 37°. Under these conditions we only expect photoemission from the top surface of the copper grid. Our laser fluences are below the ablation limit of copper^69^. Scanning electron microscopy images show no damage after prolonged laser exposure at ~20 mJ/cm^2^ (see SI10).

Our setup is equipped with a feedback system to stabilize the pump and probe laser beams onto the sample and the photocathode, respectively. The system consists of several cameras together with piezo-motorized mirrors and a home-built controlling software. For the pump beam the camera continuously records an image that contains two laser spots picked off from two beam samplers (leakage from back-polished mirrors) at different positions and the computer program determines the laser spot positions by Gaussian fits at a rate of 2 Hz. It then uses the motors to adjust the beam pointing to keep it at a fixed position. The same camera system is used to calibrate the laser spot size at the TEM sample position. The calibration was done by inducing two laser damage spots on a gold-coated Quantifoil^TM^ grid, which were well-separated with a measurable distance (by TEM) by moving the laser beam with the piezo motors and monitoring the motion of the laser spot on the camera. This allows us to correlate a real-space distance in the TEM sample plane with a number of camera pixels, yielding 500 nm/pixel. From this, we estimate the laser spot size to be 22 × 22 µm^2^ (FWHM), which translates into a footprint of 22 × 36 µm^2^ on the sample after adjustment for the known laser incident angle. The laser beam center position standard deviation is less than two pixels over 1 s of measurement. Uncertainty in the laser fluence (~1 mJ/cm^2^) is caused by beam position jitter that is faster than the camera measurement rate, variations in optics reflectivity after the laser power measurement, and pump pulse energy instabilities (few %).

Images are normalized to their total integrated intensity in order to compensate for slight variations in the probe electron intensity. A median filter of 5 × 5 pixels is applied to the images to mitigate random noise (the detector has 2000 × 2000 pixels). Difference images were generated by subtracting an averaged pre-time zero image from all subsequent frames. We also subtracted a frame recorded without probe electrons, but with pump laser beam in order to remove pump laser scatter that reaches the detector. Circular ROI radii were chosen with the goal of simultaneously optimizing the visibility and signal-to-noise ratio.

### Analytical model

Our analytical derivations are based on the use of a Gaussian model for the charge distribution. For a non-interacting system it is a straightforward proof that the evolutions of the statistics of an ensemble of particles are independent of the spatial distribution of the particles; therefore, to most closely resemble the experimental conditions, we treat the spatial distribution as Gaussian in our analysis. The time-dependent density of the electron gas is described by

3 $$\rho \left( {r,z,t} \right) = \frac{{ - Ne}}{{\left( {2\pi } \right)^{\frac{3}{2}}}}\frac{{e^{ - \frac{{z^2}}{{2\sigma _z^2\left( t \right)}}}}}{{\sigma _z\left( t \right)}}\frac{{{\mathrm{e}}^{ - \frac{{r^2}}{{2\sigma _{\mathrm{T}}^2\left( t \right)}}}}}{{\sigma _{\mathrm{T}}^2\left( t \right)}},$$

where *σ*_T_(*t*) is defined in Eq. (1) (see SI3 for derivation). The *N*-body simulation results indicate that *σ*_*z*_(*t*) becomes significantly larger than *σ*_T_(*t*) within 150 ps, which means at each time the charge distribution can be approximated by an infinitely long charged cylinder,

4 $$\rho \left( {r,t} \right) \approx \frac{{ - Ne}}{{\left( {2\pi } \right)^{\frac{3}{2}}}}\frac{1}{{\sigma _z\left( t \right)}}\frac{{{\mathrm{e}}^{ - \frac{{r^2}}{{2\sigma _{\mathrm{T}}^2\left( t \right)}}}}}{{\sigma _{\mathrm{T}}^2\left( t \right)}},$$

whose electric field can be obtained from Maxwell–Gauss law as (see SI3 for derivation)

5 $$\varepsilon _r\left( {r,t} \right) = \frac{{ - Ne}}{{\left( {2\pi } \right)^{\frac{3}{2}}{\it{\epsilon }}_0}}\frac{1}{{\sigma _z\left( t \right)}}\frac{{1 - {\mathrm{e}}^{ - \frac{{r^2}}{{2\sigma _{\mathrm{T}}^2\left( t \right)}}}}}{r}.$$

For *r* < *σ*_T_

6 $$\varepsilon _r\left( {r {\,}< {\,}\sigma _{\mathrm{T}},t} \right) \approx \frac{{ - Ne}}{{2\left( {2\pi } \right)^{\frac{3}{2}}{\it{\epsilon }}_0}}\frac{1}{{\sigma _z\left( t \right)}}\frac{r}{{\sigma _{\mathrm{T}}^2\left( t \right)}},$$

i.e. the transverse electric field is linear in the radial coordinate *r*. A linear electric field imparts a lensing effect on the probe electrons. Further derivations that lead to Eq. (2) are provided in the SI Section 2.

### Numerical simulations

We start our *N*-body simulations with a very oblate (2 × 2 × 0.01 μm^3^) 3D Gaussian slab containing 10^4^ electrons. The oblate electron slab is placed at a distance of 30 nm away from the copper surface before starting the simulations. The photoemission process itself is not included in this approach, but the rapid photoemission of the electrons renders the longitudinal dimension of the bunch very small, resulting in a pancake-like bunch after the photoemission process is complete. We assume such a bunch for the initial conditions of our simulations. The initial velocity distribution is a Gaussian with a mean of (0, 0, 6 × 10^5^ m/s) in the (*x, y, z*) directions and an isotropic spread with a standard deviation of 6 × 10^5^ m/s in each axis. These values were chosen to approximately match the velocity spread obtained from the experimental data, as well as to avoid that all electrons are absorbed by the grid. Since the copper grid is grounded, the potential at its surface is zero. Therefore, there is an electric dipole field formed between the photoemitted electron gas and its positive image charge, which is mostly aligned parallel to the propagation direction of the probe electrons. Image charges are approximated by calculating the dipole field for each electron and its positive counter-charge located at the opposite site of the copper grid surface, i.e. the electron coordinates are mirrored at the sample plane to find the coordinates of the image charges. Finally, the copper grid is approximated as a plane surface neglecting the effects of the holes. The measured cyclotron frequencies (GHz) correspond to centimeter wavelengths, which is much larger than the hole size of the grid (~4 μm), which justifies the use of a homogeneous slab instead of a grid in the simulations. An electron that hits the conductive copper plate is absorbed and excluded from the simulation. The tilt of the grid is neglected. The Lorentz force **F** = *q*(**E** + **v** × **B**) is calculated for each electron in the gas, and the equation of motion is solved using the finite difference method. A time step of 80 fs was chosen such that reducing its value further would not change the results considerably. It is assumed that the electrons move much slower than the speed of light and hence it is not necessary to use retarded electric fields or account for losses due to electromagnetic radiation.

The effect of the photoemitted electron gas on the probe electrons is simulated by placing 11025 electrons equally spaced on a square grid with 14 μm sides, centered on the optical axis, and starting their motion at 3*σ*_*z*_(*t*) above the copper grid. The kinetic energy of the probe electrons is 300 keV, corresponding to a speed of *v*_*z*_=2.3 × 10^8^ m/s (or 0.77*c*). The detector was placed at 1 m below the electron gas, and a ROI circle radius of 7 μm was used to plot the difference intensity traces. Except for the electron gas lens, no other lenses inside the TEM are considered. The comparison between experimental and simulated data is therefore only qualitative. We neglect Coulomb interactions between probe electrons, as well as any perturbations of the electron gas by the probe electrons.

## Supplementary information

Supplementary Information

Description of Additional Supplementary Files

Supplementary Movie 1

Supplementary Movie 2

Supplementary Movie 3

Supplementary Movie 4

Supplementary Movie 5

Supplementary Movie 6

Supplementary Movie 7

## Data Availability

The data generated and/or analyzed, and computer codes developed during this study are available from the corresponding author upon request.
