# Supplementary material for: Transient lensing from a photoemitted electron gas imaged by ultrafast electron microscopy
Source: Nat Commun. 2020 Jun 12;11:3001. doi: 10.1038/s41467-020-16746-z (PMC7293293; doi:10.1038/s41467-020-16746-z)
Supplement: Supplementary file 1 — Supplementary Information [file 41467_2020_16746_MOESM1_ESM.pdf]

Supplementary Information

Transient lensing from a photoemitted electron gas imaged by ultrafast electron microscopy

Omid Zandi[1,2], Allan E. Sykes[1,2], Ryan D. Cornelius[1,2], Francis M. Alcorn[1,2], Brandon Zerbe[3], Phillip M. Duxbury[3], Bryan W. Reed[4], Renske M. van der Veen[1,2,5]*

[1] Department of Chemistry, University of Illinois at Urbana-Champaign, Urbana, IL 61801, USA

[2] Materials Research Laboratory, University of Illinois at Urbana-Champaign, Urbana, IL 61801, USA

[3] Department of Physics and Astronomy, Michigan State University, East Lansing, MI 48824, USA

[4] Integrated Dynamic Electron Solutions, Inc. (IDES), Pleasanton, CA 94588, USA

[5] Department of Materials Science and Engineering, University of Illinois at Urbana-Champaign, Urbana, IL 61801, USA

* Corresponding author: renske@illinois.edu

1 Magnetic field data from the manufacturer

In Fig. S1a we compare the magnetic field that is determined in this study using the cyclotron resonance frequency and the cyclotron formula $B = 2\pi m_e/eT$, with the magnetic field for three different objective lens currents (OLC) obtained from the TEM manufacturer (courtesy Hitachi High-Tech). The data is fitted with linear functions that pass through (0,0). The slopes differ by ~5%, which could be due to a frequency shift induced by the self-Coulomb field in the electron cloud [1] or due to slight differences between lens models or microscopes. Fig. S1b shows the magnetic field as a function of z-coordinate obtained from the manufacturer. The field is uniform for a region ±1 mm away from the eucentric height, where the sample is placed, at $z = 0$.

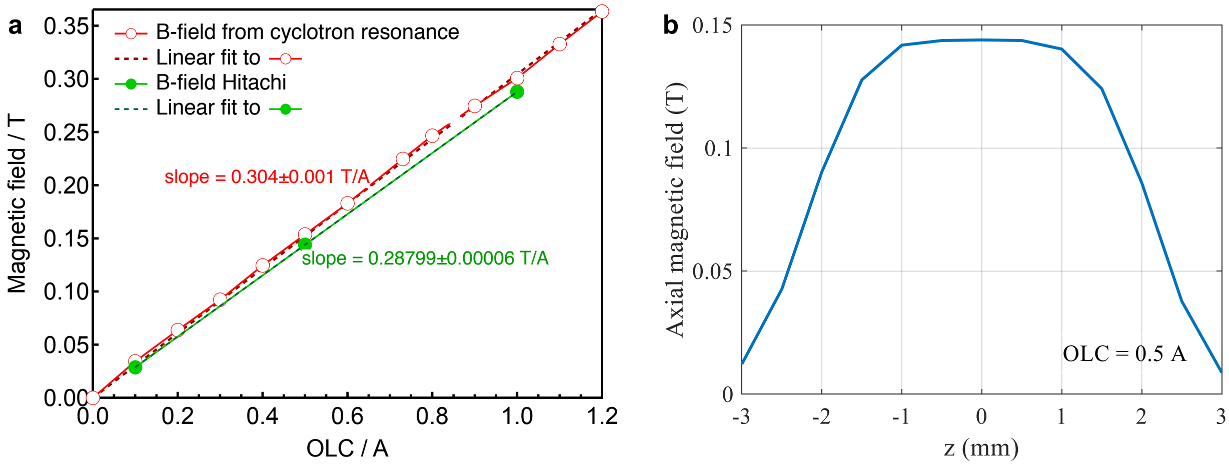

Figure S1: Magnetic field data. a Comparison between the magnetic field obtained from the measured cyclotron resonances (red circles) and the one from the manufacturer (courtesy Hitachi High-Tech) (green circles). The dashed lines are linear fits to the data (through the origin). **b** Axial magnetic field as a function of *z*-coordinate (courtesy Hitachi High-Tech).

2 Dependence on the tilt angle

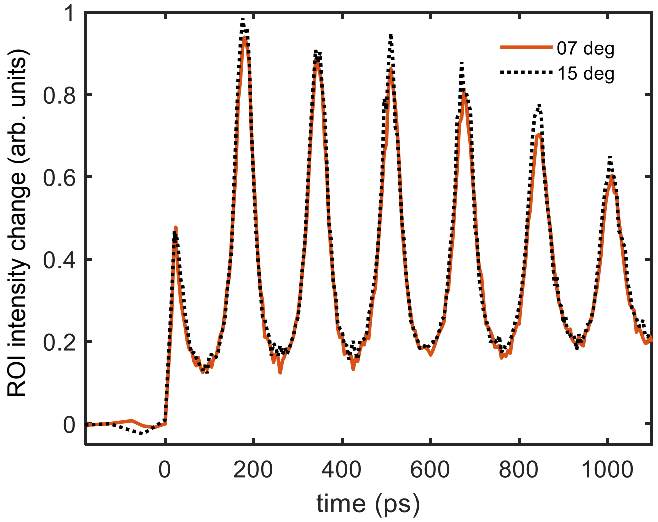

Figure S2: **Tilt dependence.** ROI difference intensity as a function of the tilt angle of the sample. The tilt direction is towards the pump laser beam. OLC = 0.7 A, ILC = 1.1 A.

3 Analytical derivations

(1) <u>Derivation of the equation for $\sigma_T(t)$ (Eq. (1) in main text):</u>

Consider a single electron created at time $t = 0$ at position $\mathbf{R} = x_0\hat{\mathbf{x}} + y_0\hat{\mathbf{y}} + z_0\hat{\mathbf{z}}$ with an initial velocity $\mathbf{v_0} = v_{x0}\hat{\mathbf{x}} + v_{y0}\hat{\mathbf{y}} + v_{z0}\hat{\mathbf{z}}$ inside a uniform time-invariant magnetic field of $\mathbf{B} = B_0\hat{\mathbf{z}}$ (Fig. S2). Applying the Lorentz force, the momentum of the electron will be obtained from

$$\frac{d\mathbf{p}}{dt} = -e\,\mathbf{v} \times B_0\hat{\mathbf{z}} = -e\big(v_x\hat{\mathbf{x}} + v_y\hat{\mathbf{y}}\big) \times B_0\hat{\mathbf{z}} = eB_0\big(v_x\hat{\mathbf{y}} - v_y\hat{\mathbf{x}}\big). \quad \text{(S1)}$$

Assuming $\mathbf{p} = \gamma m\mathbf{v}$, we will have

$$v_x = v_0\cos(\omega t + \phi)\,;\;\; v_y = v_0\sin(\omega t + \phi), \quad \text{(S2)}$$

where

$$\omega = \frac{eB_0}{\gamma m_e};\;\;\; v_0\cos(\phi) = v_{x0};\; v_0\sin(\phi) = v_{y0}. \quad \text{(S3)}$$

And the *x* and *y* coordinates of the electron will be

$$x = \frac{v_0}{\omega}\sin(\omega t + \phi) + x_0 - \frac{v_0}{\omega}\sin(\phi)\,;\;\; y = -\frac{v_0}{\omega}\cos(\omega t + \phi) + \frac{v_0}{\omega}\cos(\phi) + y_0. \quad \text{(S4)}$$

The transverse distance between the electron at time t from its initial position $\mathbf{R}$ will be

$$r(t) = \frac{v_0}{\omega}\sqrt{(\sin(\omega t + \phi) - \sin(\phi))^2 + (-\cos(\omega t + \phi) + \cos(\phi))^2} = \sqrt{2}\frac{v_0}{\omega}\sqrt{1 - \cos(\omega t)}.$$

(S5)

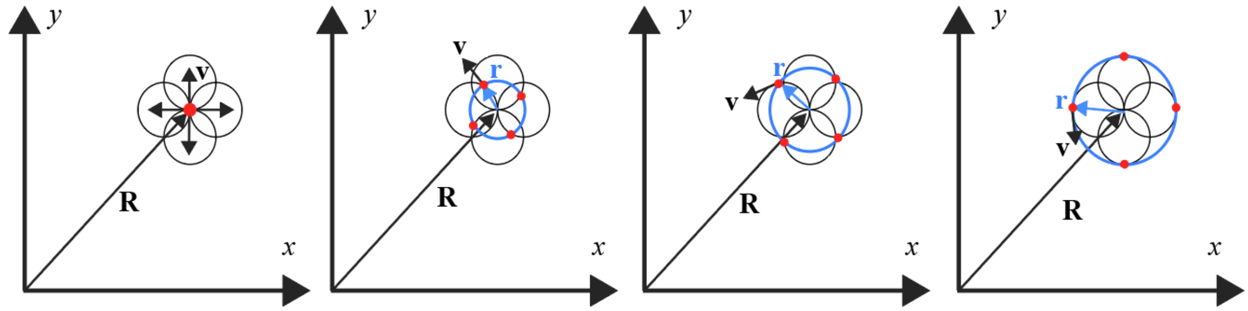

Figure S3: Position of $N = 4$ electrons at four different times. The initial velocities of the electrons have the same amplitude and a uniform angular distribution.

Now, we consider *N* electrons as shown in Fig. S3 with a normal distribution in *z*. Then, if the initial electron velocities of the *N* electrons have a uniform transverse angular distribution, their charge density will be

$$\rho_{x_0,y_0,v_0}(x,y,z,t) = -Ne\frac{e^{-\frac{z^2}{2\sigma_z^2(t)}}}{(2\pi)^{1/2}\sigma_z(t)}\frac{\delta\left(\sqrt{(x-x_0)^2+(y-y_0)^2}-\sqrt{2}\frac{v_0}{\omega}\sqrt{1-\cos(\omega t)}\right)}{2\pi\sqrt{2}\frac{v_0}{\omega}\sqrt{1-\cos(\omega t)}}, \quad \text{(S6)}$$

where $\delta(\cdot)$ is the Dirac delta function. Suppose the initial velocity amplitudes have a zero-centered Gaussian distribution of

$$f_v(v_0) = \frac{1}{2\pi\sigma_v^2}e^{-\frac{v_0{}^2}{2\sigma_v^2}}, \quad \text{(S7)}$$

with σ_v being the standard deviation of the electrons' velocities. Therefore, integration of Eq. (S6) over $f_v(v_0)$ gives

$$\rho_{x_0,y_0}(x,y,z,t) = \int_0^{\infty} 2\pi v_0\, \rho_{x_0,y_0,v_0}(x,y,z,t)\, f_v(v_0) dv_0 =$$

$$= -Ne\frac{e^{-\frac{z^2}{2\sigma_z^2(t)}}}{(2\pi)^{3/2}\sigma_z(t)}\int_0^{\infty}\frac{\delta\left(\frac{\sqrt{(x-x_0)^2+(y-y_0)^2}}{\frac{\sqrt{2}}{\omega}\sqrt{1-\cos(\omega t)}}-v_0\right)}{2\left(\frac{\sigma_v}{\omega}\right)^2(1-\cos(\omega t))}e^{-\frac{{v_0}^2}{2\sigma_v^2}}dv_0$$

$$= -Ne\frac{e^{-\frac{z^2}{2\sigma_z^2(t)}}}{(2\pi)^{3/2}\sigma_z(t)}\frac{e^{-\frac{(x-x_0)^2+(y-y_0)^2}{4\left(\frac{\sigma_v}{\omega}\right)^2(1-\cos(\omega t))}}}{2\left(\frac{\sigma_v}{\omega}\right)^2(1-\cos(\omega t))}.$$

(S8)

The electrons have an initial spatial distribution of

$$f(x_0,y_0) = \frac{1}{(2\pi)^2\sigma_x\sigma_y}e^{-\frac{{x_0}^2}{2\sigma_x^2}-\frac{y_0^2}{2\sigma_y^2}}, \quad \text{(S9)}$$

with σ_x and σ_y being the standard deviations of the zero-centered distribution in x and y, respectively, which is determined by the laser pulse profile. Therefore, the charge density will be determined by

$$\rho(x,y,z,t) = \int_{-\infty}^{\infty}\int_{-\infty}^{\infty} \rho_{x_0,y_0}(x,y,z,t) f(x_0,y_0) dx_0 dy_0$$

$$= -Ne \frac{e^{-\frac{z^2}{2\sigma_z^2(t)}}}{(2\pi)^{3/2}\sigma_z(t)} \frac{1}{2\left(\frac{\sigma_v}{\omega}\right)^2(1-\cos(\omega t))} \frac{1}{(2\pi)^2\sigma_x\sigma_y} \int_{-\infty}^{\infty}\int_{-\infty}^{\infty} e^{-\frac{(x-x_0)^2+(y-y_0)^2}{4\left(\frac{\sigma_v}{\omega}\right)^2(1-\cos(\omega t))}} e^{-\frac{x_0{}^2}{2\sigma_x^2}-\frac{y_0^2}{2\sigma_y^2}} dx_0 dy_0$$

$$= -Ne \frac{e^{-\frac{z^2}{2\sigma_z^2(t)}}}{(2\pi)^{1/2}\sigma_z(t)} \frac{1}{2\left(\frac{\sigma_v}{\omega}\right)^2(1-\cos(\omega t))} \frac{1}{(2\pi)^2\sigma_x\sigma_y}$$

$$\times \int_{-\infty}^{\infty} e^{-\frac{(x-x_0)^2}{4\left(\frac{\sigma_v}{\omega}\right)^2(1-\cos(\omega t))}} e^{-\frac{x_0{}^2}{2\sigma_x^2}} dx_0 \int_{-\infty}^{\infty} e^{-\frac{(y-y_0)^2}{4\left(\frac{\sigma_v}{\omega}\right)^2(1-\cos(\omega t))}} e^{-\frac{y_0^2}{2\sigma_y^2}} dy_0 \,.$$

(S10)

From the identity

$$\int_{-\infty}^{\infty} \mathrm{e}^{-(x-x_0)^2/A} \mathrm{e}^{-(x)^2/B} dx = \frac{\mathrm{e}^{-\frac{x_0{}^2}{A+B}}\sqrt{\pi}}{\sqrt{\frac{1}{A}+\frac{1}{B}}}; \text{ for } A, B \in \mathbb{R}_{>0} \qquad \text{(S11)}$$

Eq. (S10) becomes

$$\rho(x,y,z,t) = \frac{-Ne}{(2\pi)^{\frac{3}{2}}} \frac{1}{4\left(\frac{\sigma_v}{\omega}\right)^2(1-\cos(\omega t))} \frac{e^{-\frac{z^2}{2\sigma_z^2(t)}}}{\sigma_x\sigma_y\sigma_z(t)}$$

$$\times \frac{\mathrm{e}^{-\frac{x^2}{4\left(\frac{\sigma_v}{\omega}\right)^2(1-\cos(\omega t))+2\sigma_x^2}}}{\sqrt{\frac{1}{4\left(\frac{\sigma_v}{\omega}\right)^2(1-\cos(\omega t))}+\frac{1}{2\sigma_x^2}}} \frac{\mathrm{e}^{-\frac{y^2}{4\left(\frac{\sigma_v}{\omega}\right)^2(1-\cos(\omega t))+2\sigma_y^2}}}{\sqrt{\frac{1}{4\left(\frac{\sigma_v}{\omega}\right)^2(1-\cos(\omega t))}+\frac{1}{2\sigma_y^2}}} .$$

(S12)

If $\sigma_v/\omega > \sigma_x, \sigma_y$, the transverse profile of the electron cloud will be mostly determined by the electron velocity distribution rather than the laser profile. Therefore, for the sake of simplicity, we assume that $\sigma_x = \sigma_y = \sigma_r$ and for $r^2 = x^2 + y^2$, we find

$$\rho(r,z,t) = \frac{-Ne}{(2\pi)^{\frac{3}{2}}} \frac{e^{-\frac{z^2}{2\sigma_z^2(t)}}}{\sigma_z(t)} \frac{\mathrm{e}^{-\frac{r^2}{2\sigma_\mathrm{T}^2(t)}}}{\sigma_\mathrm{T}^2(t)}, \tag{S13}$$

where

$$\sigma_\mathrm{T}(t) = \sqrt{2\left(\frac{\sigma_v}{\omega}\right)^2 (1-\cos(\omega t)) + \sigma_r^2}\ , \tag{S14}$$

and $\sigma_z(t)$ increases linearly by time after the Coulomb explosion regime. Eq. (S14) is the same as Eq. (1) in the main text.

(2) Use of the Maxwell-Gauss law to obtain the electric field from the charge density:

When $\sigma_z(t) \gg \sigma_\mathrm{T}(t)$ the charge distribution can be approximated by an infinitely long charged cylinder, and we may write

$$\rho(r,t) \approx \frac{-Ne}{(2\pi)^{\frac{3}{2}}} \frac{1}{\sigma_z(t)} \frac{\mathrm{e}^{-\frac{r^2}{2\sigma_\mathrm{T}^2(t)}}}{\sigma_\mathrm{T}^2(t)}\ , \tag{S15}$$

whose electric field can be obtained from the Maxwell-Gauss law by

$$\mathcal{E}_r(r,t) = \frac{-Ne}{(2\pi)^{\frac{3}{2}}\epsilon_0} \frac{1}{\sigma_z(t)} \frac{1-\mathrm{e}^{-\frac{r^2}{2\sigma_\mathrm{T}^2(t)}}}{r}\ . \tag{S16}$$

For $r < \sigma_\mathrm{T}$, the first term of the Taylor expansion of Eq. (S16) is

$$\mathcal{E}_r(r<\sigma_\mathrm{T},t) \approx \frac{-Ne}{2(2\pi)^{\frac{3}{2}}\epsilon_0} \frac{1}{\sigma_z(t)} \frac{r}{\sigma_\mathrm{T}^2(t)}\ . \tag{S17}$$

(3) Derivation of the focal length equation using the linear-field approximation and impulse on probe electrons (Eq. (2) in main text):

The electron probe equation of motion in the transverse direction is

$$\frac{dp_r}{dt} = -e\mathcal{E}_r(r,t) \quad \text{for} \quad |t - t_0| < \frac{\tau_\mathrm{p}}{2} \ , \tag{S18}$$

where p_r is the transverse momentum of the probe electron, t_0 is the time the probe electron arrives at the sample plane, $\mathcal{E}_r$ is the transverse component of the electric field and τ_p is the time that the probe electron traverses the sample area and can be approximated by

$$\tau_\mathrm{p} = \frac{\sigma_z(t)}{v_z} \ , \tag{S19}$$

where $\sigma_z(t)$ is the std of the electron cloud in the z direction and v_z the probe electron velocity. Solving Eq. (S18) gives

$$\Delta p_r = p_r - p_{r0} = -e\int_{t_0-\frac{\tau_\mathrm{p}}{2}}^{t_0+\frac{\tau_\mathrm{p}}{2}} \mathcal{E}_r(r,t)dt \approx -e\frac{\sigma_z(t)}{v_z}\mathcal{E}_r(r,t_0) \ , \tag{S20}$$

where p_{r0} is the initial momentum of the electrons and indeed represents the divergence of the probe electron beam determined by its brightness (at the TEM condenser stage). For an almost parallel beam, we set $p_{r0} = 0$. Solving for transverse displacement, we have

$$\Delta r = r - \frac{e\sigma_z(t_0)}{\gamma v_z m_\mathrm{e}}\mathcal{E}_r(r,t_0)(t - t_0) = r - \frac{e\sigma_z(t)}{\gamma v_z^2 m_\mathrm{e}}\mathcal{E}_r(r,t_0)(z - z_0) \ , \tag{S21}$$

where, as is shown in Fig. (S3), Δr is the time-dependent transverse distance of the probe electrons from the optical axis, and r is the initial transverse distance. Inserting the electric field from Eq. (S17) into Eq. (S21) gives

$$\Delta r = r - \frac{e\sigma_z(t_0)}{\gamma v_z m_\mathrm{e}}\mathcal{E}_r(r,t_0)(t - t_0) = r + \frac{Ne^2}{2(2\pi)^{\frac{3}{2}}\epsilon_0\gamma v_z^2 m_\mathrm{e}}\frac{r}{\sigma_\mathrm{T}^2(t)}(z - z_0). \tag{S22}$$

Solving Eq. (S22) for $\Delta r = 0$ gives the focal length of the electron gas as

$$f_\mathrm{EG} = -\frac{2(2\pi)^{\frac{3}{2}}\epsilon_0\gamma v_z^2 m_\mathrm{e}}{Ne^2}\sigma_\mathrm{T}^2(t), \tag{S23}$$

which is Eq. (2) in the main text.

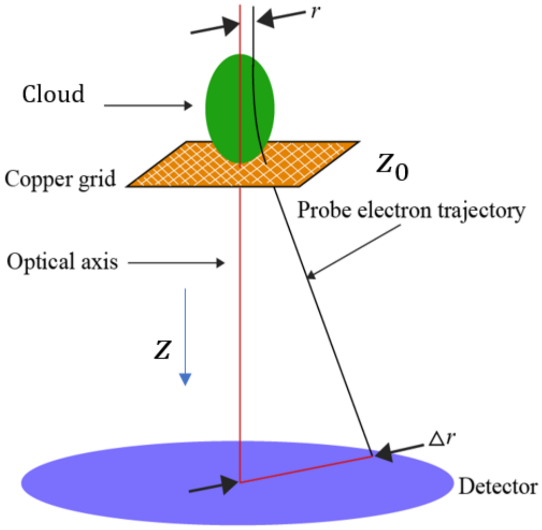

Figure S4: **Schematic of the probe-electron deflection by the electron cloud.** The cloud (green) is centered around the optical axis, and the copper grid is placed at z_0. The lateral probe-electron deflection at position z is given by Δr.

(4) Derivation of relationship between ΔS_{ROI} and electron-gas focal length:

Suppose we replace all the lenses after the cloud (sample plane) by a single lens whose focal length is f_{EL} and we call it the equivalent lens (EL). In the thin lens approximation, the total focal length of the lensing system (equivalent lens and electron-gas lens) is

$$f_{\text{total}}(t) = \frac{f_{\text{EL}} f_{\text{EG}}(t)}{f_{\text{EL}} + f_{\text{EG}}(t) - d}, \tag{S24}$$

where d is the distance between the cloud and the EL. We assume d (cm) is negligible in comparison to $f_{\text{EG}}(t)$ (m). Fig. S5 shows the simplified imaging system where probe electrons with the initial radius r_i pass through the lens and hit the detector.

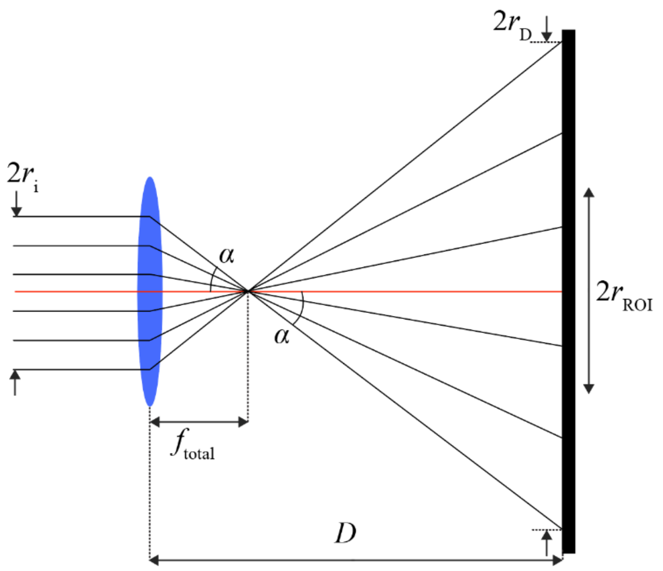

Figure S5: Lens model. A simplified model of the lensing system that represents the cloud lens plus a lens equivalent to all the TEM lenses after the sample.

According to Fig. S4

$$\tan\alpha = \frac{r_i}{f_{total}} = \frac{r_D}{D - f_{total}} \rightarrow r_D = \frac{r_i}{f_{total}}(D - f_{total}), \tag{S25}$$

where r_D is the radius of the probe beam on the detector and D is the distance between the lensing system and the detector. The density of the probe electrons on the detector is

$$\rho_D = \frac{N_p}{\pi r_D^2}\ , \tag{S26}$$

where N_p is the number of probe electrons. The detected intensity in the ROI is

$$S_{ROI} = 2\pi\int_0^{r_{ROI}} \rho_D r dr = N_p \frac{r_{ROI}^2}{r_D^2} = N_p \frac{r_{ROI}^2}{r_i^2 (D - f_{total})^2} f_{total}^2. \tag{S27}$$

By assuming $D \gg f_{total}$, for a high excitation of the intermediate lens (>1 A), we have

$$S_{ROI} \approx \frac{N_p r_{ROI}^2}{r_i^2 D^2} f_{total}^2\ . \tag{S28}$$

If we subtract the pre-time zero signal, we will get

$$\Delta S_{ROI} \approx N_p \frac{r_{ROI}^2}{r_i^2 D^2}\left(f_{total}^2 - f_{EL}^2\right). \tag{S29}$$

Then

$$\Delta S_{\mathrm{ROI}} \approx N_{\mathrm{p}} \frac{r_{\mathrm{ROI}}^2}{r_{\mathrm{i}}^2 D^2} \left(\frac{f_{\mathrm{EL}} f_{\mathrm{EG}}(t)}{f_{\mathrm{EL}} + f_{\mathrm{EG}}(t) - d} + f_{\mathrm{EL}} \right) \left(\frac{f_{\mathrm{EL}} f_{\mathrm{EG}}(t)}{f_{\mathrm{EL}} + f_{\mathrm{EG}}(t) - d} - f_{\mathrm{EL}} \right)$$

$$\approx -N_{\mathrm{p}} \frac{r_{\mathrm{ROI}}^2 f_{\mathrm{EL}}^2}{r_{\mathrm{i}}^2 D^2} \left(2 - \frac{f_{\mathrm{EL}} - d}{f_{\mathrm{EG}}(t)} \right) \left(1 - \frac{f_{\mathrm{EL}} - d}{f_{\mathrm{EG}}(t)} \right) \frac{f_{\mathrm{EL}} - d}{f_{\mathrm{EG}}(t)} \approx -2N_{\mathrm{p}} \frac{r_{\mathrm{ROI}}^2 f_{\mathrm{EL}}^2}{r_{\mathrm{i}}^2 D^2} \frac{f_{\mathrm{EL}} - d}{f_{\mathrm{EG}}(t)}, \tag{S30}$$

where we have assumed $f_{\mathrm{EG}}(t) \gg f_{\mathrm{EL}} - d$ (i.e. the electron-gas lens is much weaker than the TEM projection lenses). Therefore, if we excite the intermediate lens strongly, while the effect of the cloud lensing is still observable, the detected signal in the region of interest is approximately inversely proportional to the focal length of the cloud.

We note that this description of the lens system is highly simplified. It serves as a phenomenological model that gives us the right scaling in the focal length and the number of electrons (see below). Future work will focus on making this treatment more quantitative by considering the divergence of the incoming electron beam, and the distances between and excitation of all TEM lenses. In addition, geometric effects, such as the rather large dimension of the electron cloud along the *z*-direction, also need to be taken into account in order to reach quantitative agreement.

(5) Estimation of the number of electrons in the cloud:

From Eq.'s (S16) and (S17), the cloud radial electric field maximizes when $\sigma_{\mathrm{T}}(t)$ is minimum at $\omega t = 2n\pi; n = 0,1,2, \ldots$ for which the amount of probe electron deflection by the cloud depends only on N and its minimum transverse size σ_r. From Eq. (S22), the number of electrons is

$$N = 2(2\pi)^{\frac{3}{2}} \epsilon_0 \gamma v_z^2 \frac{m}{e^2} \frac{\sigma_r^2}{(z_d - z_0)} \left(\frac{\Delta r}{r} \bigg|_{\omega t = 2n\pi} - 1 \right), \tag{S31}$$

where $\frac{\Delta r}{r}\Big|_{\omega t=2n\pi}$ can be regarded as the highest magnification caused by the cloud and can be calculated as is shown in Fig. S6 and $z_d - z_0 = 100$ cm is the approximate distance between the cloud and the detector. The number of electrons then becomes ~2×10^5. Because of all the approximations, we expect this method to give us the correct order of magnitude in the number of generated electrons.

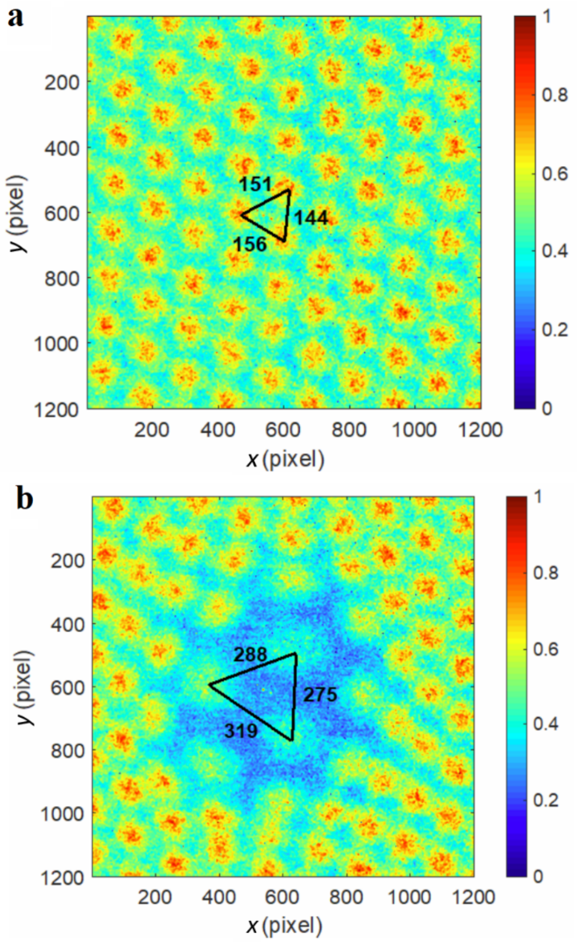

Figure S6: Magnification in the copper-grid image. a a pre-time-zero image, and **b** an image at the second peak (first cyclotron resonance). A maximum magnification of ~2 is derived. The centers of the grid holes are found by fitting Gaussians.

4 Details of the fit of the ROI difference intensity trace

Eq. (S30) suggest that we can fit the inverse of the focal length function to the ROI signal in the focused regime with high excitation of the IL (current 1.1 A). Accordingly, we fit the function

$$1/f_{\mathrm{EG}}\,(t) = \frac{A\,e^{-(t-t_0)/\tau}}{2\left(\frac{R}{\omega}\right)^2\left(1-\cos\left(\omega(t-t_0)\right)\right)+1} \tag{S32}$$

to the ROI signal as is done in Fig. 7 in the main text. Here, A is a constant that comes from combining Eq. (S23) with Eq. (S30), τ is the damping time due to the absorption of the electrons and dephasing, t_0 is an arbitrary initial time (or $\omega t_0$is the phase of oscillation), ω is the cyclotron angular frequency, σ_v is the velocity spread and σ_r is the minimum transverse size of the cloud (all σ denote standard deviations). The amplitude A and (σ_v, σ_r) are directly correlated with each other and therefore they cannot be determined independently. We, therefore, fit the ratio $R = \sigma_v/\sigma_r$, and set $\sigma_r = 12/\sqrt{2}$ µm, which is obtained from the experimental laser spot size of ~28 µm FWHM or σ = 12 µm (geometric average of major and minor axes of elliptical footprint), and considering that the electrons are emitted through a two-photon process that scales quadratically with photon intensity. Using a non-linear least-square fitting procedure (built in the Matlab curve-fitting toolbox) for time delays 100-900 ps (i.e. passed the Coulomb explosion regime) we then obtain: $A = 1.05 \pm 0.01$ m^{-1}, $\omega = 37.97 \pm 0.01$ GHz, $\sigma_v = 4.91 \pm\ 0.01 \cdot 10^5$ m/s, $t_0 = 7.8 \pm 0.1$ ps, and $\tau = 8.2 \pm 0.2$ ns.

5 Width of the cyclotron peaks

The focal length of the electron gas is

$$f_{EG}(t) = -\frac{2(2\pi)^{\frac{3}{2}}\epsilon_0 \gamma {v_z}^2 m_\mathrm{e} \sigma_r^2}{Ne^2}\left[2\left(\frac{\sigma_v}{\omega\sigma_r}\right)^2 (1-\cos(\omega t)) + 1\right]$$

and is inversely proportional to the ROI signal $S(t)$ if the intermediate lens of the UTEM has a high enough excitation. For that case

$$S(t) \propto \frac{1}{f_\mathrm{EG}(t)} \propto \frac{A}{2\left(\frac{\sigma_v}{\omega\sigma_r}\right)^2 (1-\cos(\omega t)) + 1}.$$

The full-width-at-half-maximum (FWHM) of $S(t)$ is found by solving

$$\frac{A}{2\left(\frac{\sigma_v}{\omega\sigma_r}\right)^2 (1-\cos(\omega t)) + 1} = \frac{A}{2},$$

which results in

$$\Delta t_\mathrm{FWHM} = \frac{2}{\omega}\operatorname{acos}\left(1 - \frac{1}{2\left(\frac{\sigma_v}{\omega\sigma_r}\right)^2}\right).$$

In Fig. S7, $\omega\Delta t_\mathrm{FWHM}$ is plotted versus $\frac{\sigma_v}{\omega\sigma_r}$. For a fixed value of the OL excitation (fixed ω), the FWHM depends only on $\frac{\sigma_v}{\sigma_r}$. As the velocity spread increases for a fixed ω and σ_r, the width of the signal decreases. Assuming that the velocity spread is majorly determined by the early stage of Coulomb explosion and hence the number of electrons, the FWHM is determined mostly by the laser fluence.

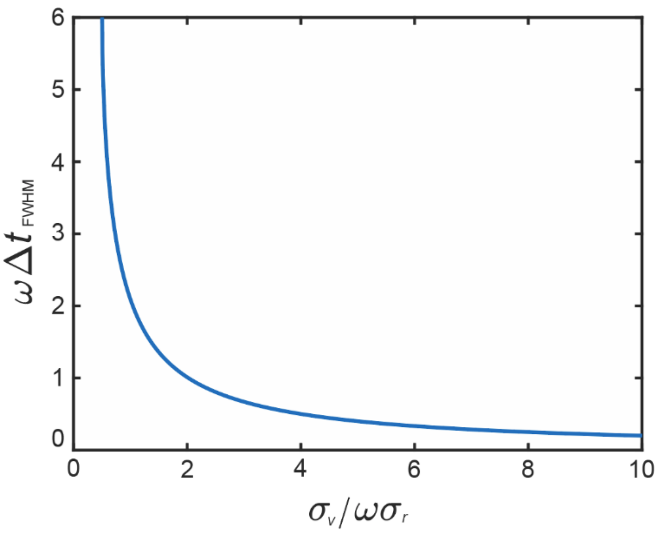

Figure S7: **Cyclotron peak widths.** Unitless FWHM of the ROI cyclotron peaks as a function of the unitless quantity $\frac{\sigma_v}{\omega\sigma_r}$.

6 Simulated absorption of electrons by the copper grid

In Fig. S8 we plot the number of electrons in the electron cloud as a function of time. The simulation starts with 10^4 electrons whose center is placed at a distance of 30 nm away from the copper grid. During the first few ps, the electron gas undergoes a Coulomb explosion due to the large density and electron-electron repulsion. This leads to a large fraction of electrons being absorbed by the grid. As expected, more electrons are absorbed when image charges are included in the simulation. At later times, the electron absorption rate decreases until the fraction of electrons left in the simulation levels off at ~50%.

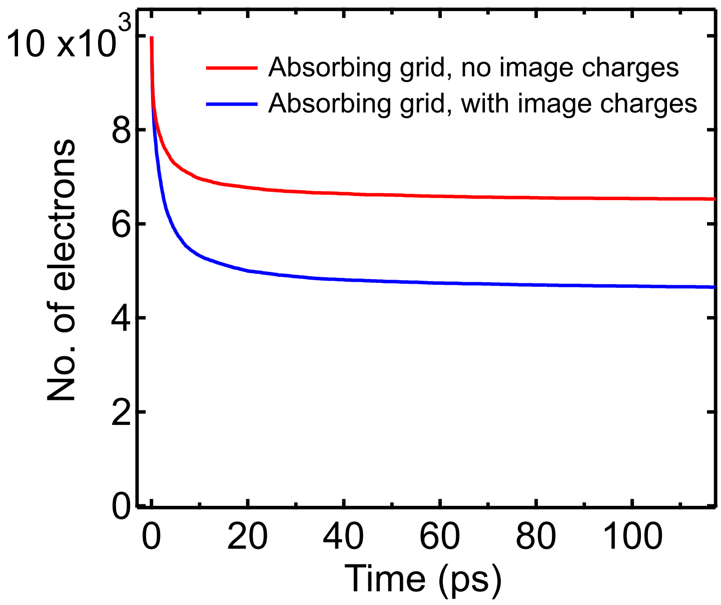

Figure S8: Electron absorption by the grid. Number of electrons in the electron gas as a function of time delay extracted from a simulation with and without image charges. A significant fraction of electrons is absorbed by the copper grid.

7 Mean kinetic energy and kinetic energy spread from simulations

Fig. S9 shows the mean kinetic energy of all electrons in the gas (**a**) and the kinetic energy spread (standard deviation) in **b** (with absorbing grid and image charges). For very early times (<1 ps), the mean kinetic energy and its spread spike to very large values (not shown) due to acceleration of the electrons towards the grid; the *y*-axis scale has been cut in order to show the data at later times. A plateau is reached around 50 ps after excitation, which indicates the regime where Coulomb interactions do not play a large role anymore.

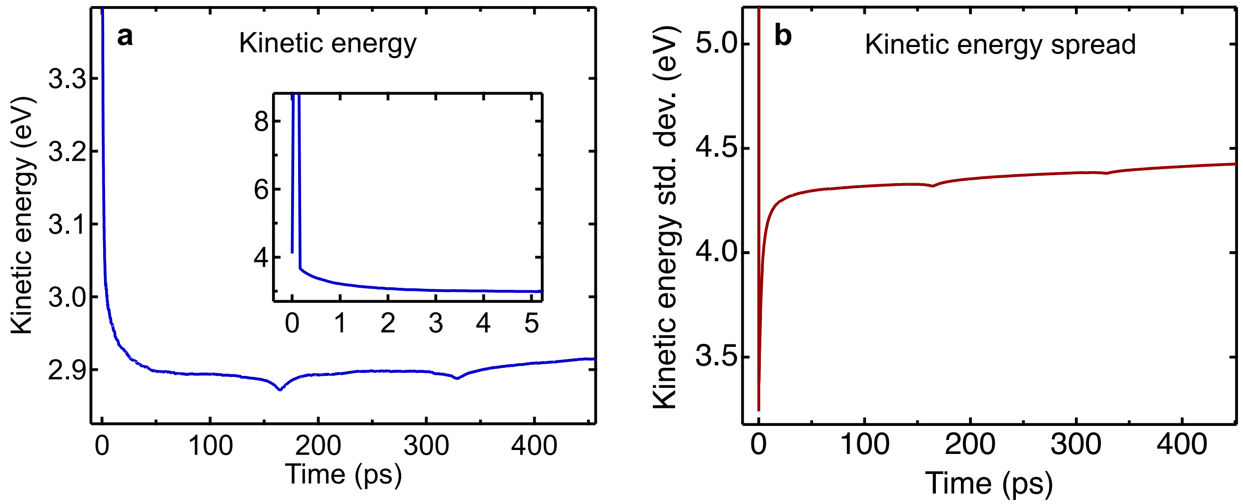

Figure S9: Mean kinetic energy and kinetic energy width. a Mean kinetic energy extracted from the simulation data. The inset shows a zoom into the early dynamics (0-5 ps). **b** Kinetic energy spread (standard deviation) energy extracted from the simulation data. Note that a fraction of electrons is absorbed by the copper grid (see Fig. S8).

8 Effect of positive image charges

Image charges are constructs to model the existence of the conductive grid to cancel the tangential component of the total electric field on the surface of a conductor. The simple image charge formalism is appropriate because the copper grid is an excellent conductor with a plasma frequency of several PHz and a thickness of many micrometers, while the simulations are on the scale of micrometers and picoseconds. Therefore, on the scale of the simulations, we may take the electric field and the charge density in the interior of the metal to be zero. Any residual bulk charges will be screened and, in effect, move to the surface before they can affect the simulation. The effective thickness of the surface charge layer will be on the nanometer scale and therefore negligible on the scale of the simulations. The metal does carry a residual positive charge, but after the initial fs-scale transient it will all reside in this extremely thin surface layer. The field of the image charges is valid only outside of the conductor [2, p. 141]. In this regard, in contrast to the electron cloud, the positive image charges do not constitute an actual charge distribution in free space. Indeed, because the grid is a grounded conductor (connected to the body of the TEM), we assume that there is no electric field below the grid (it is insulating the space below the grid from the top), as is depicted in Figure S10 below. Therefore, the probe electron that travels toward the grid does not go through two charge distributions; and any probe electron that hits the grid will be absorbed. Only those going through the holes of the grid can make it to the detector.

At early times after creation of the electron cloud, the bunch adopts a pancake-like (oblate) shape and is located very close to the Cu grid. The electric field is in this case mostly parallel to the probe electron beam and no deflection (lensing) takes place. As the electron bunch expands during the first few ps, the electron gas adopts a more prolate shape elongated along the z-axis, and the effect of the image charges is reduced since the electrostatic dipole force along z scales

with $\sim 1/d^2$ where d is the distance between the two charges. These two scenarios are depicted in Fig. S10.

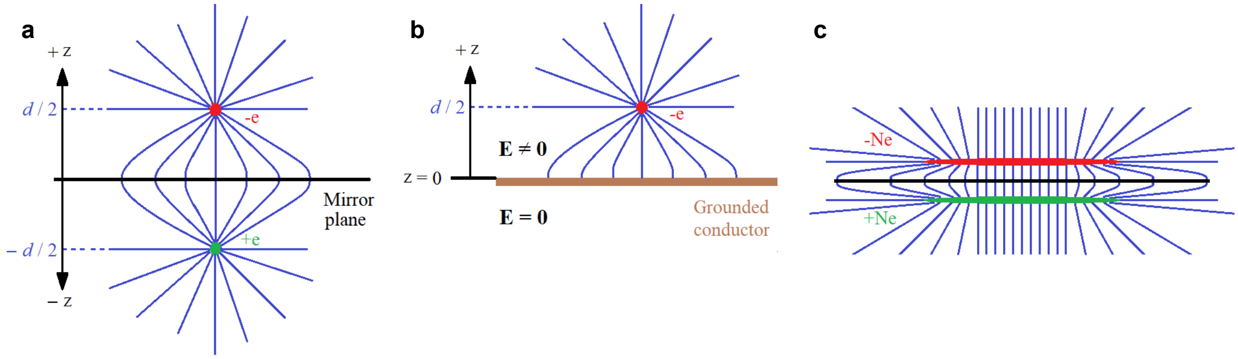

Figure S10: Positive image charges. **a** Image charge construction used to derive the electric field above a grounded conductor (copper grid) shown in **b**. As the charge moves away from the grid, the effect of the image charge reduces. Note that the grid is insulating, i.e. there is no electric field below the sample plane. **c** Schematic of the situation right after photoexcitation. The pancake-like electron bunch is very close to the grid and the electric field lines are mostly perpendicular to the sample grid and therefore parallel to the probe electrons. This explains the delay in lensing signal at very early times after excitation (first few ps).

9 Simulations of ROI intensity traces

In Fig. S11 we show a set of simulated ROI difference intensity traces extracted from *N*-body probe simulations (see main text for details) for three cases: (1) without a grid (no absorption of electrons, no image charges); (2) with an absorbing grid, but without image charges; (3) with grid and image charges. It is clear that the majority of the amplitude reduction of the first peak is coming from the image-charge effect, which includes the increased absorption of electrons due to the dipole field between electrons and image charges (see Figs. S8 and S10). Fig. S11b shows a zoom into the first tens of ps after photoexcitation. It is seen that the rise time of the ROI intensity depletion signal is prolonged in the case of image charges, even though the creation process of the electron cloud was not explicitly included in the simulation. The latter could prolong the rise time even further.

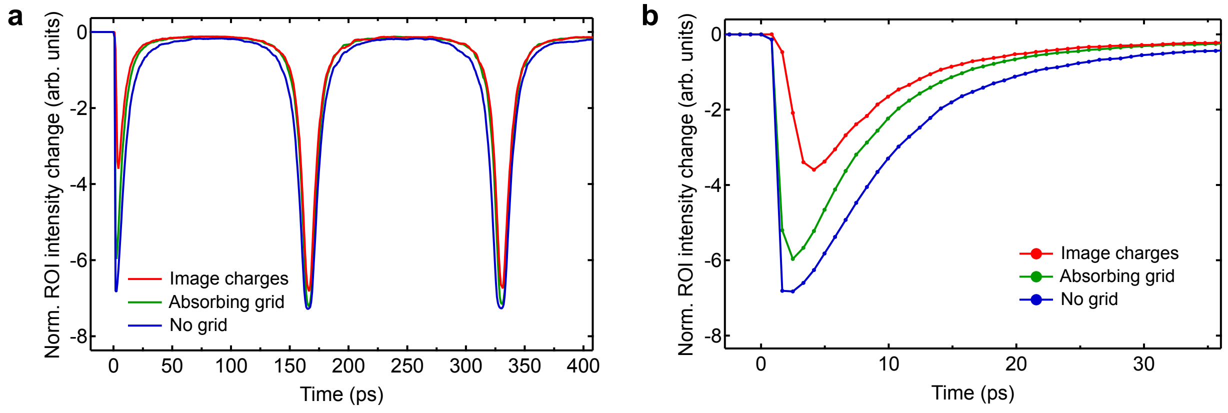

Figure S11: ROI intensity simulations. a Simulations of the ROI difference intensity obtained from the *N*-body simulations. **b** Same data as in **a**, but zoomed into the first 35 ps.

10 SEM images of copper grid

Fig. S12 shows scanning electron microscopy (SEM) images of a 200 mesh copper grid, one taken before laser exposure and one taken after ~8 h of exposure at 528 nm, ~200 fs, ~20 mJ/cm^2. No damage is seen within the resolution of the images.

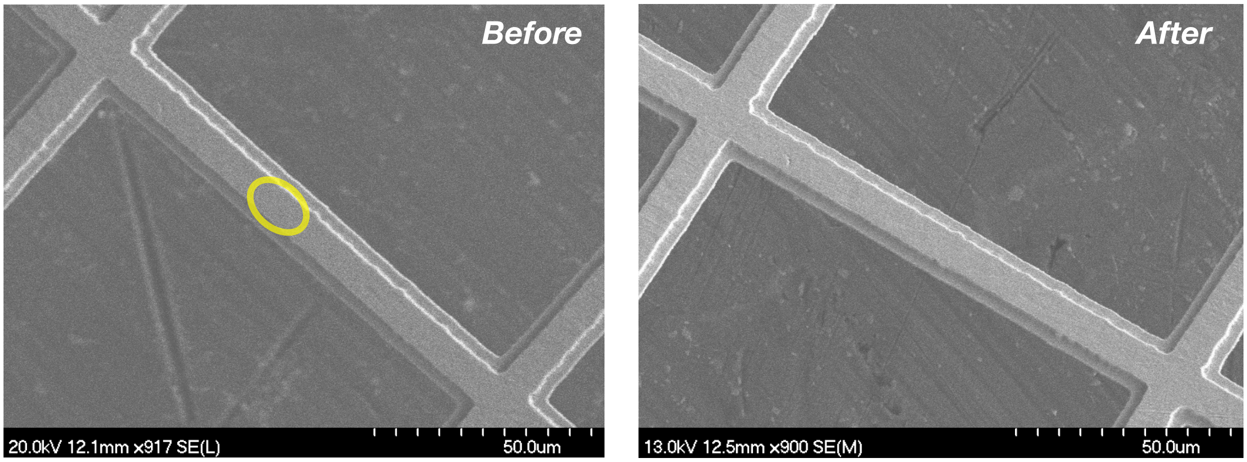

Figure S12: SEM images. Left: 200 mesh copper grid before laser exposure. Right: 200 mesh copper grid before laser exposure (528 nm, ~200 fs, 20 mJ/cm^2). The approximate irradiation area is indicated by a yellow ellipse in the left figure.

References

[1] Omid Zandi, Renske M. van der Veen, “Coulomb Explosion of an Electron Gas in a Uniform Magnetic Field”, preprint at https://arxiv.org/abs/2003.00611 (2020).

[2] David, K. Cheng. "Field and wave electromagnetics." Addison-Wesley publications (1983).
