# Supplementary material for: Transient lensing from a photoemitted electron gas imaged by ultrafast electron microscopy
Source: Nat Commun. 2020 Jun 12;11:3001. doi: 10.1038/s41467-020-16746-z (PMC7293293; doi:10.1038/s41467-020-16746-z)
Supplement: Supplementary file 2 — Description of Additional Supplementary Files [file 41467_2020_16746_MOESM2_ESM.pdf]

Description of Additional Supplementary Files

File Name: Supplementary Movie 1
Description: Difference-image movie belonging to Fig. 2a

File Name: Supplementary Movie 2
Description: Full-image movie belonging to Fig. 2b

File Name: Supplementary Movie 3
Description: Full-image movie belonging to Fig. 4b

File Name: Supplementary Movie 4
Description: Full-image movie belonging to Fig. 4c

File Name: Supplementary Movie 5
Description: Astigmatism movie belonging to Fig. 5a-c

File Name: Supplementary Movie 6
Description: N-body 3D numerical simulation movie

File Name: Supplementary Movie 7
Description: N-body numerical simulation probe movie
